# Acute HDM exposure shows time-of-day and sex-based differences in the severity of lung inflammation and circadian clock disruption

**DOI:** 10.1016/j.jacig.2023.100155

**Published:** 2023-07-24

**Authors:** Ashokkumar Srinivasan, Allan Giri, Santhosh Kumar Duraisamy, Alexander Alsup, Mario Castro, Isaac Kirubakaran Sundar

**Affiliations:** Division of Pulmonary, Critical Care and Sleep Medicine, Department of Internal Medicine, University of Kansas Medical Center, Kansas City, Kan

**Keywords:** Airway inflammation, allergic asthma, circadian rhythms, cytokine/chemokine gating, house dust mite, *Rev-erbα*KO

## Abstract

**Background:**

Asthma is a chronic inflammatory disease that shows a time-of-day response to variations in symptoms/severity. However, how the lung circadian clock influences time-of-day response and sex-based differences in house dust mite (HDM)-induced airway inflammation and remodeling has not been thoroughly investigated.

**Objective:**

We sought to determine whether acute HDM exposure in wild-type mice shows time-of-day response and sex-based differences in allergic airway inflammation and circadian clock disruption in the lungs.

**Methods:**

Wild-type (C57BL/6J) and *Rev-erbα* knockout (KO) mice were exposed to either PBS or HDM (for 10 days) intranasally at Zeitgeber time (ZT0: 6 am; ZT12: 6 pm) and euthanized 48 hours after the last exposure. Acute HDM-induced time-of-day response and sex-based differences in lung inflammation, gated cytokines/chemokines, humoral and hormonal responses, and circadian clock gene expression were analyzed.

**Results:**

Acute HDM-exposed mice showed a time-of-day response and sex-based differences in exaggerated lung inflammation (inflammatory eosinophils and interstitial macrophages) at ZT12 when compared with ZT0. HDM-exposed female mice showed increased inflammatory response at ZT12, but HDM-exposed male mice showed comparatively lower inflammation with no time-of-day response. HDM-exposed female and male mice showed augmented IgE levels at ZT12 when compared with ZT0. Myeloid innate immunity panel, cytokines/chemokines, and mucin genes showed a time-of-day gating response at ZT0 and ZT12 in the HDM group. In addition, HDM exposure altered the expression of circadian clock genes in the lung, which was evident in female mice at ZT12. Overall, female mice showed significant time-of-day responses to all these parameters compared with male mice. *Rev-erbα* KO mice exposed to acute HDM showed exaggerated lung inflammation associated with increased IgE and proinflammatory cytokines in bronchoalveolar lavage fluid. Interestingly, HDM exposure causes reduced expression of clock genes in flow-sorted resident eosinophils but not alveolar macrophages. Acute HDM exposure reduced the nocturnal locomotor activity in mice 5 days post–HDM exposure until day 10.

**Conclusions:**

This study shows a time-of-day response to acute HDM exposure and sex-based differences in the severity of lung inflammation and humoral immune response associated with circadian clock disruption. Our findings support the use of separate female and male mice cohorts for preclinical studies to understand the molecular heterogeneity in asthma pathophysiology.

Asthma is a heterogeneous disease characterized by wheezing, coughing, shortness of breath, chest tightness, and variable expiratory airflow limitation, and it affects more than 25 million adults and children in the United States and 260 million people globally.^1^ Response to medications for asthma is quite variable, and intrinsic biologic differences may explain this heterogeneity.^2^ Asthma is triggered by a plethora of allergens, one of which is house dust mite (HDM), a major perennial human allergen that exaggerates immune response associated with inflammation, airway hyperresponsiveness, mucus hypersecretion, and remodeling.^3^ Accumulating evidence shows that 75% of patients with asthma experience recurring symptoms more frequently in the early hours of the morning, with most death-related to asthma attacks reported at the same time.^1^^,^^4^ Circadian variation in lung function is a well-known phenomenon that contributes to asthma pathobiology. Innate circadian rhythms that operate under the influence of the circadian clock are known to regulate the immune system and their involvement in the rhythmic recruitment of immune cells into the lung.^4^^,^^5^

Circadian rhythms in cells and tissues appear to be synchronized via the sympathetic nervous system that orchestrates tissue-specific oscillations in immune cell recruitment. They are generated and maintained at the molecular level by clock proteins that are expressed in virtually every cell. The central clock is located in the suprachiasmatic nucleus of the brain, which synchronizes clocks in peripheral tissues via humoral and neuronal mediators. The circadian clock machinery controls the rhythmic expression of CLOCK (circadian locomotor output cycles kaput) and BMAL1 (brain and muscle ARNT-like 1). The CLOCK:BMAL1 heterodimer promotes the expression of PERs (periods 1-3) and CRYs (cryptochromes 1-2) and 2 families of nuclear receptors, the REV-ERBα/β and the RORs (retinoid-related orphan receptors). PER/CRY forms a heterodimer and translocates to the nucleus to inhibit the CLOCK:BMAL1 complex, and thus represses its own transcription. However, REV-ERBs compete with RORs to inhibit BMAL1 expression by binding to ROR response elements.^6^^,^^7^ Detailed circadian clock machinery in the lung has been reviewed recently.^4^ There is strong evidence that supports fluctuations in lung function and immune cell homing under the influence of the circadian clock, which partly explains the time-of-day difference in asthmatic symptoms.^8^^,^^9^ These rhythmic changes are greatly exacerbated in patients with nocturnal asthma. Studies suggest that acute inflammation due to Salmonella and influenza virus infection at a specific time of day affects the circadian clock machinery and its rhythmicity at the molecular level.^10^^,^^11^ Clinical studies have also shown variation in asthma severity depending on time-of-day allergen exposure.^12^ Furthermore, evidence now suggests that differential microRNA expression and altered clock proteins during chronic lung diseases such as asthma may be contributing factors in the pathobiology of the disease.^4^ Apart from the time-of-day exposure, sex-based differences have been shown to play a crucial role in asthma pathophysiology. Studies including human subjects suggest that females and males show a distinct clinical phenotype to allergen-induced immune response.^13^^,^^14^ Epidemiological reports support these sex-based differences showing an increased incidence of asthma in males during early childhood (<5 years) and in females during their adolescence.^13^^,^^14^

The present study aimed to determine whether acute HDM exposure in wild-type (WT) mice shows time-of-day response and sex-based differences in allergic airway inflammation and circadian disruption in the lungs. There are no studies to date that address sex-based differences and time-of-day response to acute HDM-induced lung inflammation and circadian clock disruption using a mouse model. For the first time, we show sex-based differences in lung immune-inflammatory response and humoral response associated with the time-of-day HDM exposure in mice. We found that acute HDM exposure at Zeitgeber time 12 (ZT12) had profound effects on the recruitment of immune-inflammatory cells into the lungs in female mice compared with male mice. Our study shows that acute HDM-induced exaggerated inflammation during the active phase (ZT12) has a profound effect on lung circadian clock disruption, augmenting asthmatic lung phenotype including altered humoral response and cytokine/chemokine gating in mice.

## Methods

### Mice

Adult female and male C57BL/6J WT mice (stock no. 000664) and *Rev-erbα* KO mice (strain no. 018447), about 2 to 3 months old, were obtained from Jackson Laboratory (Bar Harbor, Me) and housed in a regular 12:12 light-dark cycle at the Research Support Facility vivarium in the University of Kansas Medical Center. All the animal protocols described in this study were approved by the Institutional Animal Care and Use Committee of the University of Kansas Medical Center (ACUP no. 2020-2575).

### Acute HDM model

Adult female and male C57BL/6J mice were exposed to sterile PBS or HDM (extract [lot nos. 371587 and 315580] from Greer Laboratories, Lenoir, NC) for 10 days. In brief, mice were dosed with 30 μL PBS (control) or HDM (30 μg in 30 μL) intranasally under mild anesthesia using 5% isoflurane at a specific time of day (ZT0: 6 am; ZT12: 6 pm) for 10 consecutive days. Mice were euthanized 48 hours after the last PBS or HDM exposure at ZT0 or ZT12 to compare the time-of-day response to acute HDM exposure (see Fig E1 in this article’s Online Repository at www.jaci-global.org). For additional details on the experimental methods, see this article’s Methods section in the Online Repository at www.jaci-global.org.

### Statistical analysis

The statistical analysis was carried out using GraphPad Prism 9 software (GraphPad, La Jolla, Calif). Differences between 2 or more experimental groups were assessed by 2-way ANOVA using the Tukey multiple comparison test. Three-way interaction analysis between treatment (PBS versus HDM), sex (female versus male), and time (ZT0 versus ZT12) was assessed using generalized linear modeling through the R statistical computing language (version 4.2.3; R Core Team 2021; R Foundation for Statistical Computing, Vienna, Austria [https://www.R-project.org/]). For each outcome measure, a generalized linear model was fit including 2- and 3-way interactions. Because of the limited sample size of the experiment and in consideration of convergence issues, this analysis is included as an Online Repository table (see Table E1 in this article’s Online Repository at www.jaci-global.org) and descriptive statistics within the figures. Results were presented as mean ± SEM. A *P* value less than .05 was considered statistically significant.

## Results

### Acute HDM exposure shows sex-based differences and time-of-day response in the lung

**Fig 1:**
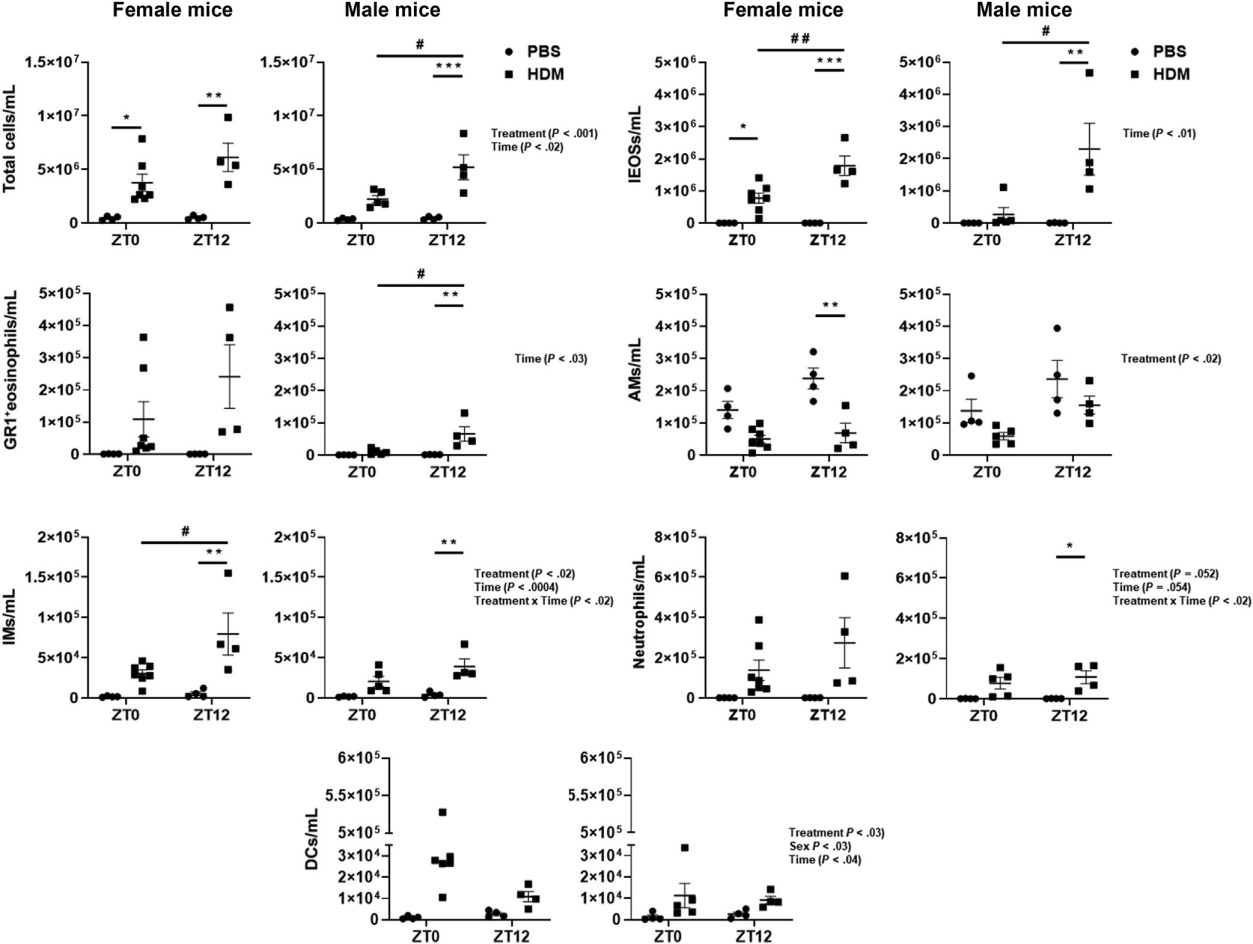
Sex differences in myeloid cell infiltration from BAL fluid show a time-of-day response to acute HDM exposure analyzed by flow cytometry. Total cells in BAL fluid were determined using Countess II FL automated cell counter (Thermo Fisher Scientific Inc, Waltham, Mass) using trypan blue staining. Myeloid cell types iEOSs, GR1^+^ EOSs, IMs, neutrophils, AMs, and DCs from BAL fluid of acute (10 days) PBS- and HDM-exposed female and male mice at ZT0 and ZT12 were analyzed by flow cytometry. Data are shown as mean ± SEM (n = 4-7/group [female mice] and n = 4-5/group [male mice]). ^∗^*P* < .05, ^∗∗^*P* < .01, and ^∗∗∗^*P* < .001, compared with respective control (PBS) at ZT0 or ZT12 in female or male mice; ^#^*P* < .05 and ^#^^#^*P* < .01, compared with the HDM group at ZT0 versus ZT12 in female or male mice.

To determine the sex-based differences and the time-of-day response (ZT0 versus ZT12) to acute HDM-induced lung inflammation, bronchoalveolar lavage (BAL) fluid and lung tissues of female and male mice were analyzed separately. Representative dot plots of the multicolor flow-cytometry panel used to identify myeloid subsets in this study are presented (see Fig E2, *A* and *B*, in this article’s Online Repository at www.jaci-global.org). Acute HDM-exposed female mice showed a significant increase in total cell counts and inflammatory eosinophils (iEOSs; CD11b^+^, Siglec F^+^, and CCR3^+^) at ZT0 and ZT12 and interstitial macrophages (IMs; CD11b^+^, IA/IE^+^, and CD11c^−^) at ZT12 but reduced alveolar macrophages (AMs; CD11c^+^, Siglec F^+^, and CD11b^−^) at ZT12 in BAL fluid compared with their respective PBS group (Fig 1). Similarly, acute HDM-exposed male mice showed a significant increase in total cell counts, iEOSs, GR1^+^ eosinophils (GR1^+^ EOSs; CD11b^+^, Siglec F^+^, and GR1^+^), IMs, and neutrophils (Ly6G/GR1^+^ and CD11b^+^) at ZT12 compared with their respective PBS group, but not at ZT0 (Fig 1). iEOSs in BAL fluid were significantly increased at ZT12 in HDM-exposed female and male mice (Fig 1). HDM-exposed female mice showed increased iEOSs at ZT0 and ZT12 compared with male mice. Similarly, GR1^+^ EOSs were increased in HDM-exposed male mice at ZT12. Although HDM-exposed female mice showed no significant difference in GR1^+^ EOSs at ZT0 and ZT12, they were relatively higher in female mice compared with male mice at ZT12 (Fig 1). AMs were higher in PBS-exposed female and male mice compared with their HDM group. However, PBS-exposed female mice showed higher AMs at ZT12 compared with their respective HDM group, and male mice showed no significant difference between PBS and HDM groups at ZT0 and ZT12. IMs were increased in HDM-exposed female and male mice at ZT12 compared with their respective PBS group, but the HDM-exposed female mice had increased IMs compared with the male mice (Fig 1). Neutrophils and dendritic cells (DCs; CD11c^+^ and IA/IE^+^) were slightly increased but did not reach significance in HDM-exposed female mice at ZT0 and ZT12 compared with their respective PBS group. However, neutrophils were significantly increased in HDM-exposed male mice at ZT12 compared with the PBS group (Fig 1). Additional interaction analysis from BAL differential cell counts data revealed statistical significance for total cells (Treatment and Time), iEOSs and GR1^+^ EOSs (Time), AMs (Treatment), IMs and neutrophils (Treatment, Time, and Treatment × Time), and DCs (Treatment, Sex, and Time) (Fig 1 and Table E1).

**Fig 2:**
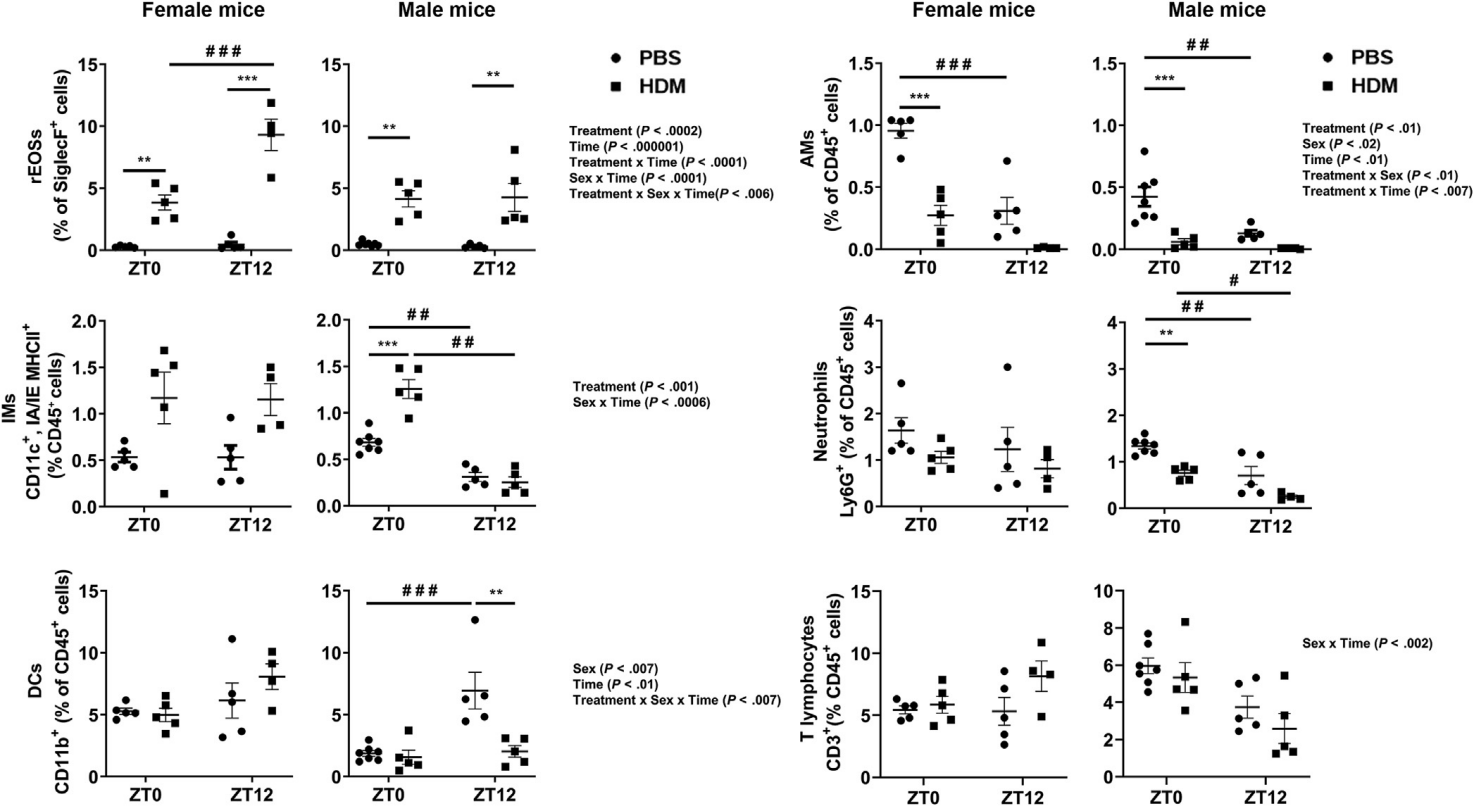
Sex differences in myeloid cell infiltration in lung tissues show a time-of-day response to acute HDM-induced lung inflammation analyzed by flow cytometry. Total cells in dissociated lung tissues were determined using Countess II FL automated cell counter using trypan blue staining. Myeloid cell types rEOSs, AMs, IMs, neutrophils, DCs, and T lymphocytes from lung tissue of acute (10 days) PBS- and HDM-exposed female and male mice at ZT0 and ZT12 were analyzed by flow cytometry. Data are shown as mean ± SEM (n = 4-5/group [female mice] and n = 4-7/group [male mice]). ^∗∗^*P* < .01 and ^∗∗∗^*P* < .001, compared with respective control (PBS) at ZT0 or ZT12; ^#^*P* < .05, ^#^^#^*P* < .01, and ^#^^#^^#^*P* < .001, compared with the PBS or HDM group at ZT0 versus ZT12.

Acute HDM-exposed female mice showed a significant increase in rEOSs at ZT0 and ZT12, an increasing trend of IMs at ZT0 and ZT12, and a significant reduction in AMs at ZT0 in lung tissues compared with their respective PBS groups (Fig 2). Similarly, acute HDM-exposed male mice showed a significant increase in rEOSs at ZT0 and ZT12 and in IMs at ZT0 when compared with their respective PBS groups. AMs and neutrophils in acute HDM-exposed male mice were significantly reduced at ZT0 compared with the PBS group, but not at ZT12 (Fig 2). However, HDM-exposed female mice showed a significant time-of-day response to exaggerated rEOSs at ZT12 compared with that at ZT0 (Fig 2). AMs in PBS-exposed female and male mice were significantly reduced at ZT0 compared with those at ZT12, but not for the HDM-exposed group at ZT0 and ZT12. IMs in HDM-exposed male mice were significantly increased at ZT0 compared with the PBS group but remained unchanged at ZT12 for the PBS and HDM groups (Fig 2). Neutrophils and DCs in female mice were not significantly changed at ZT0 and ZT12 between PBS and HDM groups (Fig 2). Furthermore, neutrophils were significantly reduced in male mice that showed time-of-day response to acute HDM exposure at ZT0 and further reduced at ZT12 for PBS and HDM groups compared with the PBS or HDM group at ZT0 versus ZT12. DCs in male mice were not affected by PBS and HDM exposure at ZT0, but the PBS group at ZT12 showed significantly increased DCs compared with the PBS group at ZT0 and the HDM group at ZT12. T lymphocytes in PBS- and HDM-exposed female mice were not affected at ZT0, but HDM-exposed female mice showed a slight increase in T lymphocytes at ZT12. However, male mice showed a decreasing trend at ZT12 compared with ZT0 in PBS- and HDM-exposed groups (Fig 2). Interaction analysis of differential cells from lung tissues revealed statistical significance for rEOSs (Treatment, Time, Treatment × Time, Sex × Time, and Treatment × Sex × Time), AMs (Treatment, Sex, Time, Treatment × Sex, and Treatment × Time), IMs (Treatment and Sex × Time), DCs (Sex, Time, Treatment × Sex × Time), and T lymphocytes (Sex × Time) (Fig 2 and Table E1). In addition, H&E staining and inflammation scores revealed a significant increase in eosinophil infiltration surrounding the airways (peribronchial), blood vessels (perivascular), and alveolar region in the lungs of acute HDM-exposed mice compared with the PBS group. As expected, acute HDM-exposed mice showed a significant increase in mucus production confirmed by the periodic acid-Schiff staining scores compared with the PBS group (see Fig E3, *A* and *B*, in this article’s Online Repository at www.jaci-global.org). We did not perform a time-of-day response to PBS- and HDM-exposed female and male mice using histological analysis separately. Overall, our findings support a definite sex-based and time-of-day difference in immune-inflammatory response in the lung to acute HDM exposure in female mice at ZT12 compared with male mice that may depend on several factors including time of HDM exposure.

### Acute HDM exposure shows the time-of-day response in BAL fluid and lung tissues

To determine whether acute HDM exposure shows the time-of-day response to lung cellular infiltration in BAL fluid and lung tissues, immunophenotyping of myeloid cells was performed by flow cytometry in PBS- and HDM-exposed female and male mice combined at ZT0 and ZT12. Results revealed a significant increase in total cell counts in the HDM group at ZT12 compared with the HDM group at ZT0 because of exaggerated eosinophil infiltration (see Fig E4, *A*, in this article’s Online Repository at www.jaci-global.org). In addition, the eosinophil subtypes iEOS and GR1^+^ EOS were significantly higher at ZT12 in the HDM group but not at ZT0 in the HDM group when compared with their respective PBS groups, and between ZT12 and ZT0 for the iEOSs (Fig E4, *B* and *C*). The total number of AMs was significantly reduced at both ZT0 and ZT12 in the HDM group compared with their respective PBS group (Fig E4, *D*). IMs were higher at both ZT0 and ZT12 in the HDM group than in their respective PBS group but were significantly higher only at ZT12. Interestingly, IMs were significantly higher at ZT12 in the HDM group than at ZT0 (Fig E4, *E*). Neutrophils were higher in the acute HDM-exposed groups at both ZT0 and ZT12 but were statistically significant only at ZT12 compared with the PBS groups at ZT12. There was no significant difference in neutrophils between ZT0 and ZT12 in the HDM group (Fig E4, *F*). DCs remain unaltered at ZT0 and ZT12 in the HDM groups and also within their respective PBS groups (Fig E4, *G*). Overall, immunophenotyping analysis of BAL fluid revealed a time-of-day response in altered myeloid cell infiltration of iEOSs and IMs.

Resident eosinophils (rEOSs; CD11b^+^, Siglec F^+^, and CCR3^−^) in the lung were significantly increased at ZT12 in the HDM group compared with ZT0 in the HDM group and with their respective PBS group (see Fig E5, *A*, in this article’s Online Repository at www.jaci-global.org). Lung AMs were reduced at ZT0 and ZT12 in the HDM group when compared with the respective PBS group. Additionally, AMs were significantly higher at ZT0 in the PBS group compared with ZT0 in the HDM group and at ZT12 in the PBS group. Interestingly, there was an inverse correlation between AMs and rEOSs at ZT0 and ZT12 in the HDM group compared with the PBS group (Fig E5, *A* and *B*). IMs showed an increased influx in the lungs at ZT0 in the HDM group compared with ZT0 in the PBS group and ZT12 in the HDM group. However, there was no significant difference at ZT12 in the HDM group when compared with that at ZT12 in the PBS group (Fig E5, *C*). Neutrophils, DCs, and T lymphocytes did not show a time-of-day response to acute HDM exposure in the lungs (Fig E5, *D-F*), but DCs were significantly increased at ZT12 compared with ZT0 in the PBS group (Fig E5, *E*). Overall, these findings suggest that acute HDM-induced exaggerated immune-inflammatory response may depend on the time of allergen exposure in mice, and these differences are observed 48 hours after 10 days of acute exposure.

### Acute HDM exposure shows altered total and HDM-specific serum immunoglobulins and time-of-day response in mice

**Fig 3:**
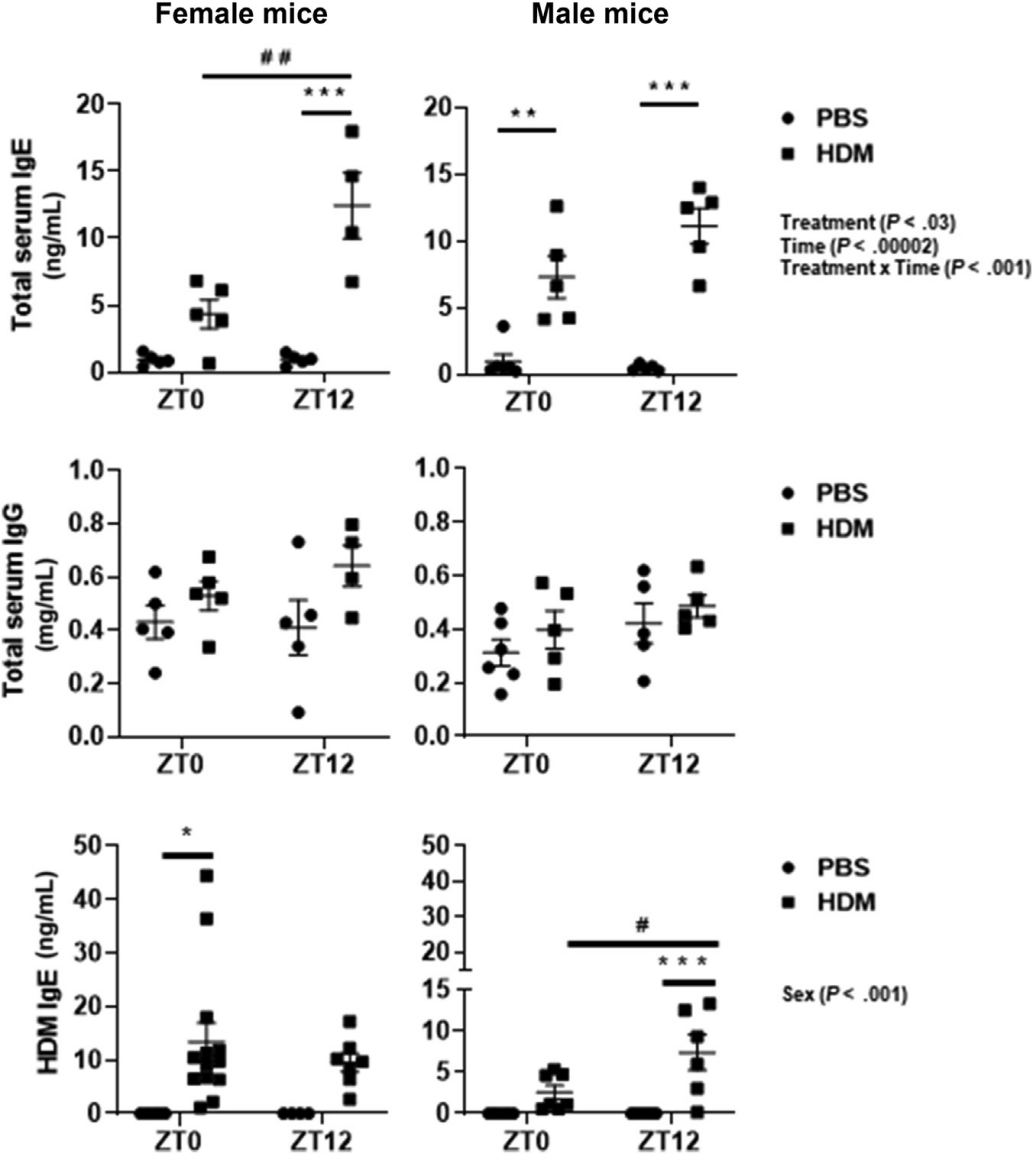
Sex-based differences in total IgE and IgG levels show a time-of-day response to acute HDM exposure. Total IgE (ng/mL) and IgG (mg/mL) levels in the serum of acute (10 days) PBS- and HDM-exposed female and male mice were determined using ELISA. Data are shown as mean ± SEM (n = 4-5/group [female mice] and n = 5-6/group [male mice]). HDM-specific IgE levels in the serum were measured from another experiment of acute (10 days) PBS- and HDM-exposed female and male mice using ELISA. Data are shown as mean ± SEM (n = 4-13/group [female mice] and n = 6-8/group [male mice]). ^∗∗^*P* < .01 and ^∗∗∗^*P* < .001, compared with respective control (PBS) at ZT0 or ZT12; ^#^^#^*P* < .01, compared with the HDM group at ZT0 versus ZT12.

To examine the acute HDM-induced difference in the humoral response, total and HDM-specific serum immunoglobulins were measured at ZT0 and ZT12. A sex-based comparison of total IgE revealed that acute HDM-exposed female mice show time-of-day response at ZT12 compared with ZT0 (Fig 3). Male mice exposed to HDM showed significantly increased total IgE at ZT0 and ZT12 compared with the respective PBS group (Fig 3). Female and male mice exposed to acute HDM did not show any change in total IgG between PBS and HDM groups at both ZT0 and ZT12 (Fig 3). Interaction analysis showed significant differences for Treatment, Time, and Treatment × Time in total serum IgE (Fig 3 and Table E1). Total IgE levels were very low in the PBS group, with no change between ZT0 and ZT12. However, the acute HDM-exposed group showed a significant increase in total IgE at ZT12 compared with ZT0 (see Fig E6, *A*, in this article’s Online Repository at www.jaci-global.org). However, total IgG showed no significant change and time-of-day response in the PBS and HDM groups at ZT0 and ZT12 (Fig E6, *B*).

There was a significant increase in HDM-specific IgE at ZT0 in acute HDM-exposed female mice compared with the PBS group but not for HDM-specific IgE at ZT0 in HDM-exposed male mice compared with the PBS group (Fig 3). Interaction analysis showed a significant difference for Sex alone in HDM-specific IgE (Fig 3 and Table E1). Interestingly, male mice showed a time-of-day response and a significant increase in HDM-specific IgE at ZT12 in the HDM-exposed group compared with the PBS group. However, female mice showed an increasing trend in HDM-specific IgE at ZT12 which was not significant. This observed difference in female and male mice for HDM-specific IgE levels at ZT0 and ZT12 measured from another experiment may be a result of batch effect (different set of mice and lot no. of HDM used) observed in this HDM-specific IgE data (because of a lack of sufficient serum samples from the same experiment).

**Fig 4:**
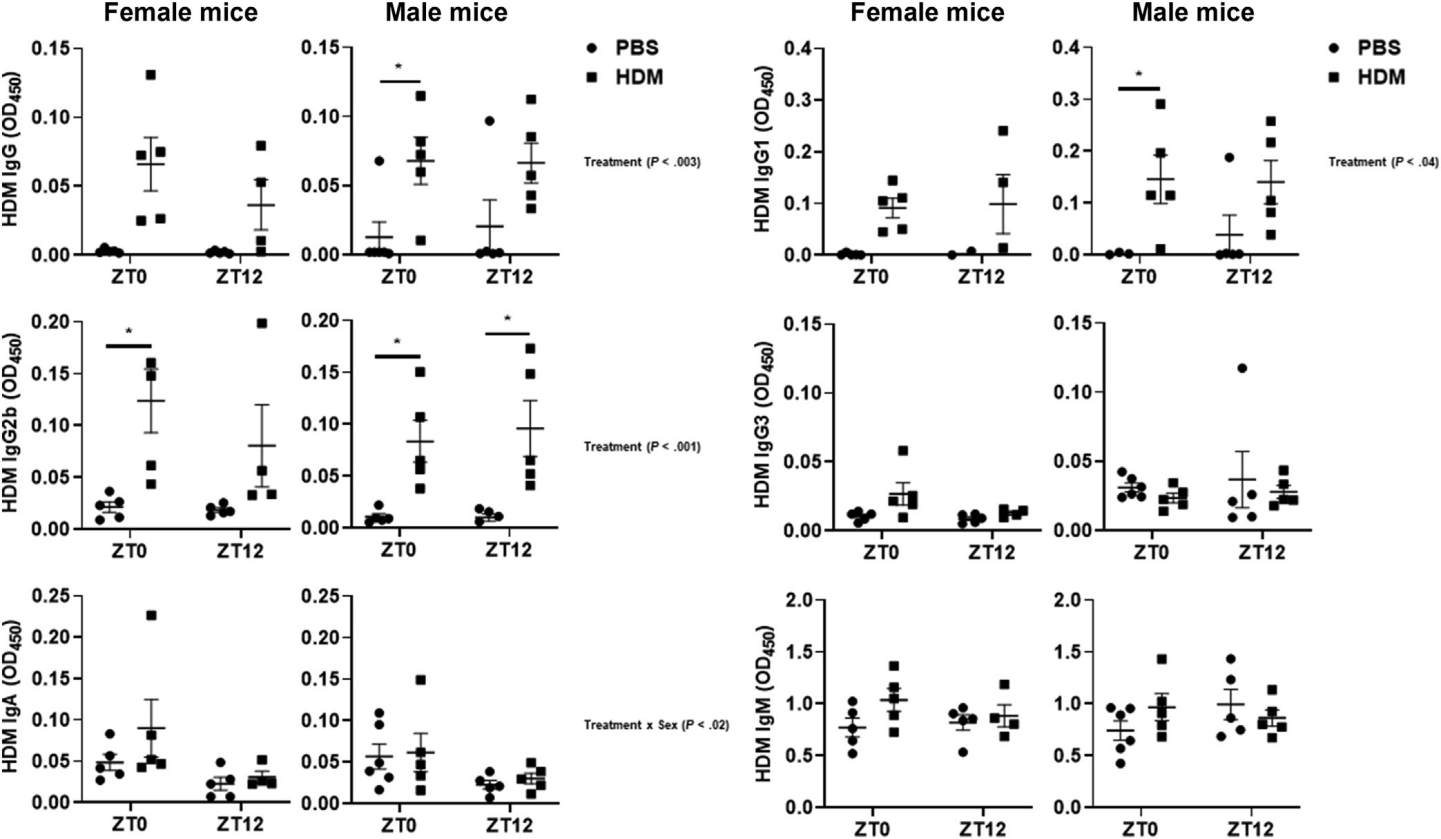
Sex-based differences in HDM-specific immunoglobulin response to acute HDM exposure. HDM-specific IgG, IgG1, IgG2b, IgG3, IgA, and IgM levels in the serum of acute (10 days) PBS- and HDM-exposed female and male mice were determined using ELISA. Data were expressed as absorbance at 450 nm. Data are shown as mean ± SEM (n = 4-5/group [female mice] and n = 5-6/group [male mice]). ^∗^*P* < .05, compared with respective control (PBS) at ZT0 or ZT12.

Sex-based comparison of HDM-specific immunoglobulins (IgG, IgG1, IgG2b, IgG3, IgA, and IgM) showed no time-of-day response in female and male mice at ZT0 and ZT12 (Fig 4). Female mice exposed to HDM at ZT0 showed a significant increase in HDM-specific IgG2b compared with ZT0 in the PBS group. HDM-specific IgG and IgG1 showed increasing trends in HDM-exposed female mice at ZT0 and ZT12 but were not significant. However, HDM-specific IgG3 and IgA showed a slight increase at ZT0 in the HDM group compared with the PBS group. HDM-specific IgM was not affected in PBS-exposed female mice versus the HDM group at ZT0 and ZT12 (Fig 4). HDM-exposed male mice showed a significant increase in HDM-specific IgG2b at ZT0 and ZT12 when compared with the respective PBS group. Similarly, HDM-exposed male mice showed a significant increase in IgG and IgG1 at ZT0 compared with the PBS group and a trend toward increased IgG and IgG1 in the HDM group at ZT12 compared with the PBS group (Fig 4). All the other HDM-specific immunoglobulins (IgG3, IgA, and IgM) remained unaffected in male mice at ZT0 and ZT12 in the PBS group versus the HDM group (Fig 4). Interaction analysis revealed statistical significance for HDM-specific IgG, HDM-specific IgG1, and HDM-specific IgG2b (Treatment). However, for HDM-specific IgA, interaction analysis showed statistical significance only for Treatment × Sex. No interaction was observed for HDM-specific IgG3 and IgM (Fig 4 and Table E1).

HDM-specific immunoglobulins IgE, IgG, IgG1, and IgG2b were increased in HDM-exposed female and male mice combined at ZT0 and ZT12 compared with their respective PBS groups. No significant difference was observed between ZT0 and ZT12 for the HDM-specific immunoglobulins (Fig E6, *C-F*). The remaining HDM-specific immunoglobulins such as IgG3, IgA, and IgM were not affected in the PBS and HDM groups at ZT0 and ZT12 (Fig E6, *G-I*). We did not observe any time-of-day response to HDM-specific immunoglobulins in acute HDM-exposed mice (Fig E6, *C-H*). Overall, we found a difference in total and HDM-specific immunoglobulins in female and male mice that may be influenced by the time of HDM exposure.

### Acute HDM exposure shows differential expression and time-of-day response to myeloid innate immunity genes in the lung

**Fig 5:**
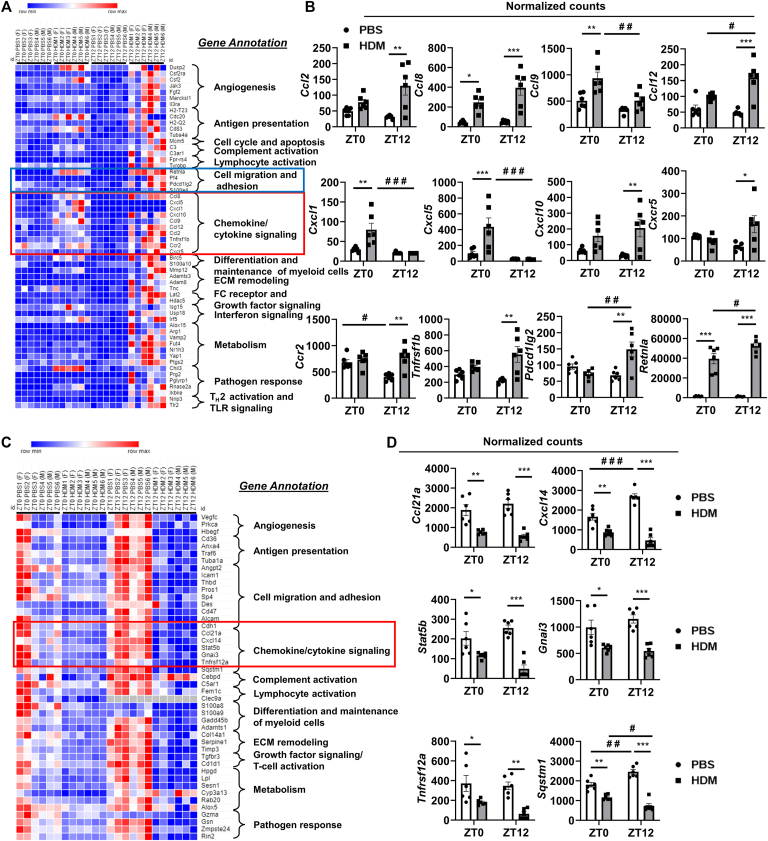
Acute HDM exposure differentially affects the gating of chemokines/cytokines, cell migration, and adhesion signaling including other myeloid innate immunity genes analyzed using NanoString. Total RNA was isolated from the lungs of acute (10 days) PBS- and HDM-exposed mice at ZT0 and ZT12. We performed NanoString analysis using a mouse myeloid immunity panel in an nCounter SPRINT Profiler (NanoString Technologies, Seattle, Wash). Normalized RNA counts were analyzed using the nSolver analysis software (version 4.0; NanoString Technologies). **A,** The heatmap generated by the Morpheus tool shows selected target genes significantly upregulated in HDM-exposed group compared with PBS control at ZT0 or ZT12. **B,** Selected chemokines/cytokines, T-cell activation, cell migration, and adhesion genes that show a time-of-day difference in the mRNA expression on the basis of normalized counts data. **C,** The heatmap generated by the Morpheus tool shows selected target genes significantly downregulated in the HDM-exposed group compared with PBS control at ZT0 or ZT12. **D,** Selected chemokine and cytokine genes that show a time-of-day difference in the mRNA expression on the basis of normalized counts data. White and gray bars in the graphs represent PBS and HDM groups, respectively, at ZT0 and ZT12. *ECM*, Extracellular matrix; *FC*, fragment crystallizable; *TLR*, Toll-like receptor. Data are shown as mean ± SEM (n = 6 [3 female + 3 male mice]/group). ^∗^*P* < .05, ^∗∗^*P* < .01, and ^∗∗∗^*P* < .001, compared with respective control (PBS) at ZT0 or ZT12; ^#^*P* < .05, ^#^^#^*P* < .01, and ^#^^#^^#^*P* < .01, compared with the PBS or HDM group at ZT0 versus ZT12.

To preferentially profile acute HDM-induced myeloid cell types in mouse lungs at ZT0 versus ZT12, we performed gene expression analysis of the myeloid innate immunity panel. We summarized our findings from PBS versus HDM groups at ZT0 and ZT12 observed to highlight chemokine/cytokine signaling, cell adhesion, and migration that show time-of-day response (Fig 5, *A* and *B*). NanoString data showed a time-of-day response and significantly increased gene expression of chemokines *Ccl2*, *Ccl8*, *Ccl12*, and *Cxcl10* that are involved in the recruitment of monocytes, DCs, and lymphocytes at ZT12 compared with the PBS group (Fig 5, *A* and *B*). However, gene expression of several chemokines was not significantly increased at ZT0 versus ZT12 in the HDM group and remained higher at ZT12 in the HDM group compared with that at ZT0 in the HDM and their respective PBS groups. In addition, the Resistin-like α (*Retnla*) gene known to play an important role in T_H_2-mediated inflammation was significantly increased at ZT0 and ZT12 in the HDM group compared with their respective PBS group. Furthermore, a time-of-day response for the *Retnla* gene expression was significantly increased at ZT12 in the HDM group compared with ZT0 (Fig 5, *A* and *B*). In addition, neutrophil-specific chemokine genes *Cxcl1* and *Cxcl5* and monocyte/macrophage-specific chemokine genes *Ccl9* and *Cxcl14* were significantly reduced at ZT12 in the HDM group compared with ZT0, showing a time-of-day response to acute HDM exposure in mice (Fig 5, *A-D*).

Gene expression of *Ccr2* was significantly decreased at ZT12 in the PBS group compared with ZT0. However, *Ccr2* expression was increased at ZT12 in the HDM group compared with the respective PBS group (Fig 5, *A* and *B*). HDM exposure at ZT12 significantly increased *Cxcr5* expression, whereas the expression remained unaltered at ZT0 in the HDM group compared with the PBS group. Genes that belong to the TNF receptor superfamily involved in inflammatory response show differential response, *Tnfrsf12a* reduced at ZT0 and ZT12, and *Tnfrsf1b* increased at ZT12 in the HDM group compared with the respective PBS group (Fig 5, *A-D*). Genes such as *Stat5b* and *Gnai3* (G protein subunit α I3) that regulate intracellular signaling to activate T cells thereby exaggerating lung inflammation were significantly reduced at ZT0 and ZT12 in the HDM group compared with their respective PBS group (Fig 5, *C* and *D*). *Pdcd1lg2* and *Sqstm1* genes were involved in T-cell tolerance, and autophagy was differentially expressed at ZT0 versus ZT12 in the HDM group. *Pdcd1lg2* was significantly increased at ZT12 in the HDM group compared with the PBS group and at ZT0 in the HDM group. *Sqstm1* was significantly decreased at ZT0 and ZT12 in the HDM group compared with the respective PBS group. However, *Sqstm1* was increased at ZT12 in the PBS group compared with ZT0, showing a time-of-day response (Fig 5, *A-D*). Several key genes belonging to novel cellular processes and pathways annotated in Fig 5, *A* and *C*, that were differentially expressed at ZT0 and/or ZT12 in the PBS group versus the HDM group were summarized (see Fig E7, *A-C*, and Fig E8, *A-C*, in this article’s Online Repository at www.jaci-global.org). Gene expression profiling revealed acute HDM exposure showing a time-of-day response on the differential expression of myeloid innate immunity genes that contribute to the altered magnitude of lung inflammation/injury.

### Acute HDM exposure shows exaggerated T_H_2 cytokine expression and time-of-day response in the lung

Acute HDM exposure significantly increased transcript levels of T_H_2 cytokines *Il4*, *Il5*, and *Il13* at ZT12 compared with their respective PBS group (but not at ZT0), and between ZT12 and ZT0 in the HDM group for *Il4* and *Il13* (see Fig E9, *A-C*, in this article’s Online Repository at www.jaci-global.org). HDM-exposed mice showed a significant increase in mRNA expression of *Ccl2* (at ZT12) and *Ccl8* (at ZT0 and ZT12) compared with their respective PBS group (Fig E9, *D* and *E*). Acute HDM-induced significant increase in *Il13* at ZT12 was associated with a significant increase in *Muc5ac* (at ZT12 in the HDM group compared with ZT12 in the PBS group) mucin gene that is responsible for mucus hypersecretion. Among the T_H_2 cytokines analyzed at the mRNA level, *Il4* and *Il13* showed a strong time-of-day response at ZT12 in the HDM group compared with ZT0 in the HDM or PBS group, which complements increased *Muc5ac* expression at ZT12 in the HDM group (Fig E9, *F*). Overall, our data support that gene expression of T_H_2 cytokines shows a time-of-day response to acute HDM-induced airway inflammation.

### Acute HDM exposure differentially affects circadian clock gene expression in the lung

**Fig 6:**
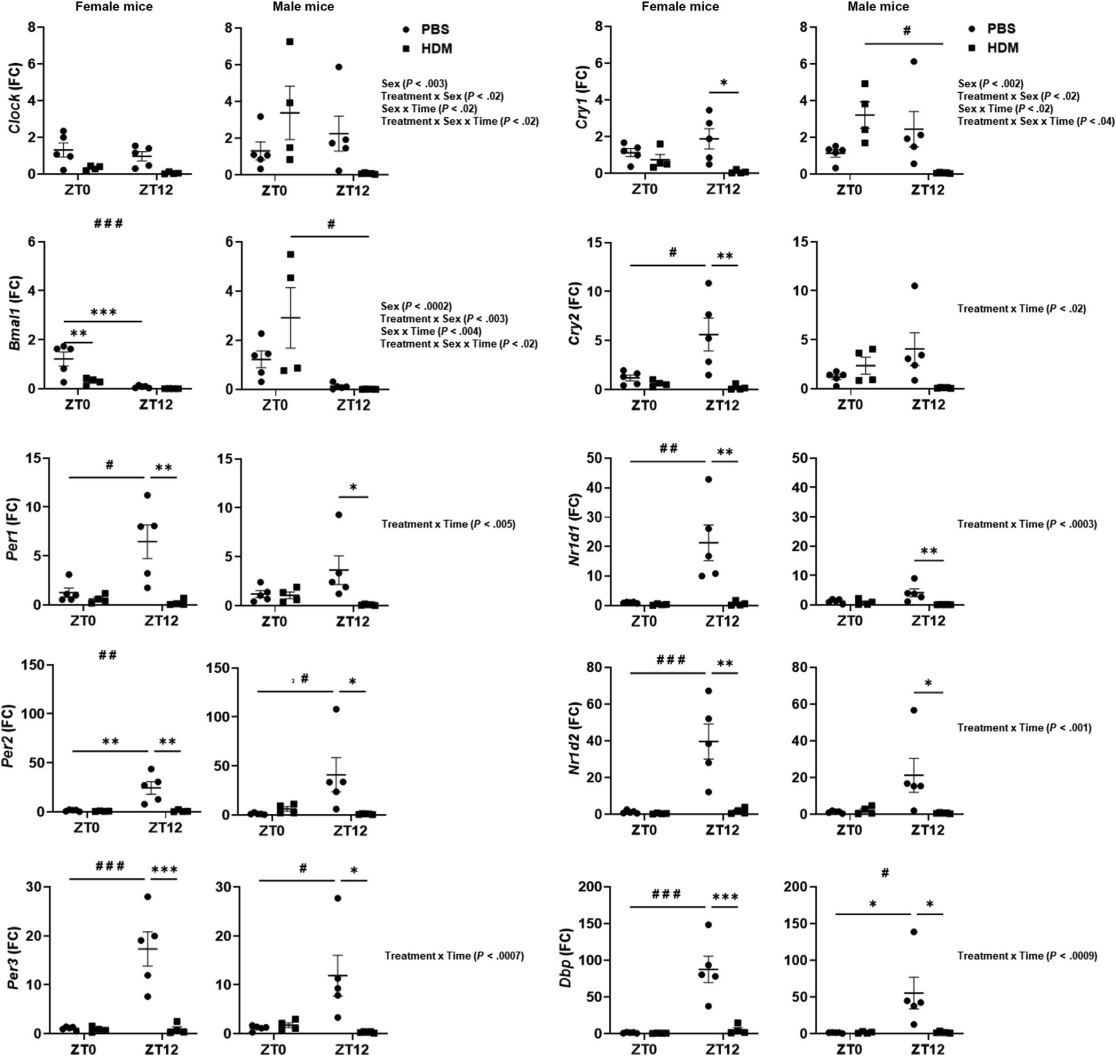
Acute HDM exposure shows a time-of-day response to circadian clock gene expression in the lungs. Total RNA was isolated from the lungs of acute (10 days) PBS- and HDM-exposed mice at ZT0 and ZT12. Gene expression of core CCGs *Clock*, *Bmal1*, *Per1*, *Per2*, *Per3*, *Cry1*, *Cry2*, *Nr1d1*, *Nr1d2*, and *Dbp* in female and male mice was determined (relative to 18S rRNA) by quantitative RT-PCR analysis and the results are presented in relation to expression changes at ZT0 in PBS group. Data are shown as mean ± SEM (n = 4-5/group [female mice] and n = 5/group [male mice]). ^∗^*P* < .05, ^∗∗^*P* < .01, and ^∗∗∗^*P* < .001, compared with respective control (PBS) at ZT0 or ZT12; ^#^*P* < .05 and ^#^^#^*P* < .01, compared with the HDM group at ZT0 versus ZT12.

To determine whether acute HDM exposure shows a time-of-day response to altered circadian clock genes, we performed quantitative RT-PCR analysis at ZT0 and ZT12 to correlate with inflammatory response and circadian clock disruption in the lungs. Interestingly, we found that *Per1*, *Per2*, *Per3*, *Nr1d1*, *Nr1d2*, and *Dpb* were significantly reduced in female and male mice at ZT12 in the HDM group compared with the respective PBS group, but not at ZT0 in the HDM group compared with the PBS group (Fig 6). *Cry1* and *Cry2* were significantly reduced in female but not in male mice at ZT12 in the HDM group compared with ZT12 in the PBS group. However, *Bmal1* was significantly reduced in female mice at ZT0 in the HDM group compared with the PBS group, but not at ZT12 in the HDM group compared with the PBS group (Fig 6). There was no significant difference between PBS- and HDM-exposed female and male mice at ZT0 for most of the core clock genes analyzed (Fig 6). Interaction analysis revealed differential responses that were statistically significant for *Clock*, *Cry1*, and *Bmal1* (Sex, Treatment × Sex, Sex × Time, and Treatment × Sex × Time). The remaining clock genes *Cry2*, *Per1*, *Per3*, *Nr1d1*, *Nr1d2*, and *Dbp* showed significant interaction only for the Treatment × Time response (Fig 6 and Table E1). In addition, the analysis of the transcript levels of core clock genes from female and male mice combined showed significantly reduced expression of *Per1*, *Per2*, *Per3*, *Cry1*, *Cry2*, *Nr1d1*, *Nr1d2*, and *Dbp* at ZT12 in the HDM group compared with ZT12 in the PBS group (see Fig E10 in this article’s Online Repository at www.jaci-global.org). We did not observe significant changes in the transcript levels at ZT0 in the PBS group and ZT0 in the HDM-exposed group. However, *Bmal1* expression was significantly reduced at ZT12 in the PBS group compared with ZT0. In contrast, all the other core clock genes (*Per1*, *Per2*, *Per3*, *Cry2*, *Nr1d1*, *Nr1d2*, and *Dbp*) were significantly increased at ZT12 in the PBS group compared with ZT0, which showed a time-of-day difference in clock gene expression in the lungs (Fig E10). Our data demonstrate a strong association of lung circadian clock disruption with time-of-day response observed in female and male mice following acute HDM exposure.

### Acute HDM exposure does not alter the serum corticosterone and serotonin levels

**Fig 7:**
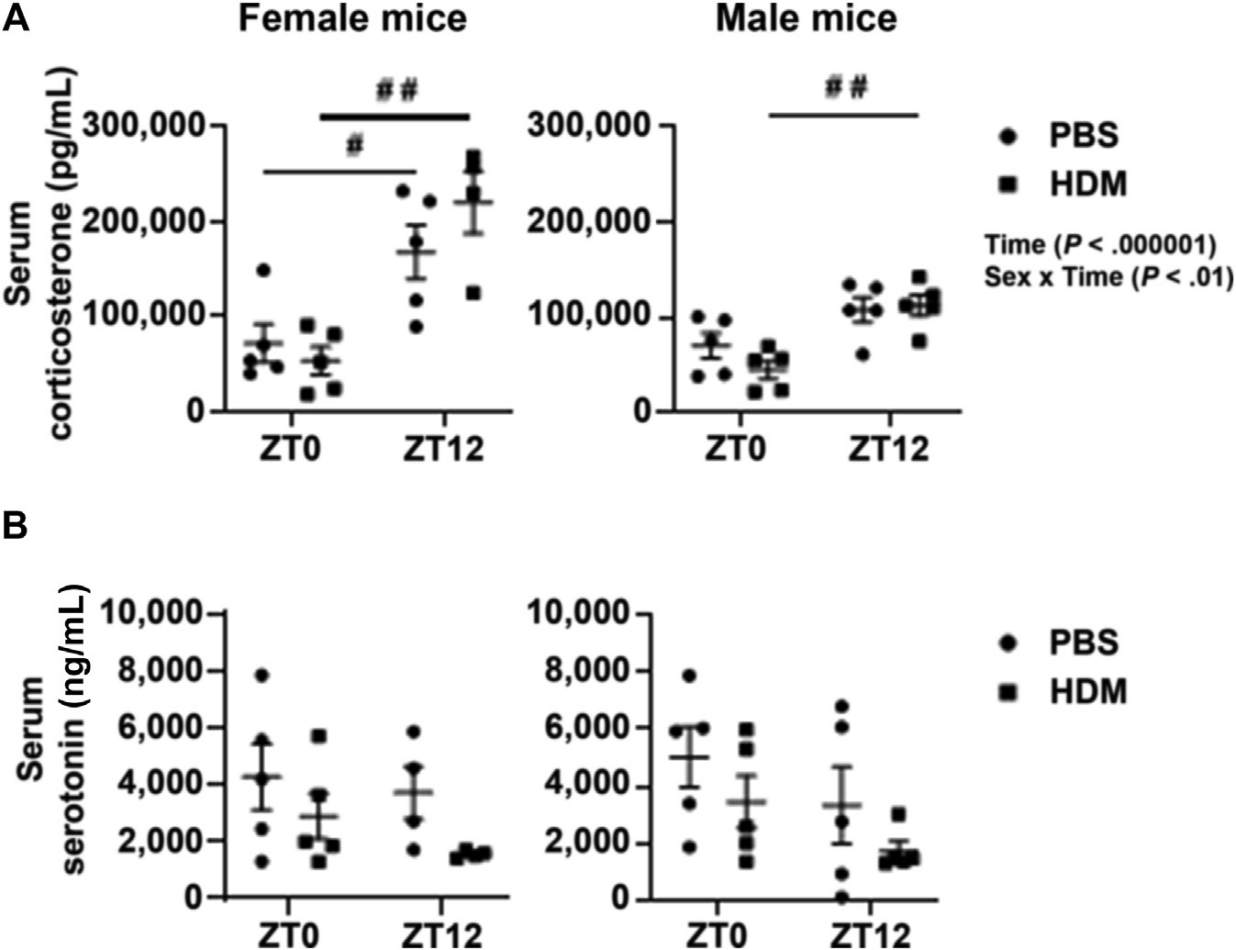
The serum stress hormone corticosterone, but not serotonin, shows a time-of-day difference in acute PBS- and HDM-exposed mice. Serum levels from C57BL/6J female and male mice exposed to acute (10 days) PBS and HDM at ZT0 and ZT12 were analyzed. Serum levels of (**A**) corticosterone (pg/mL) and (**B**) serotonin (ng/mL) were measured in PBS- and HDM-exposed female and male mice using commercially available competitive immunoassay. Data are shown as mean ± SEM (n = 4-5/group [female mice] and n = 5/group [male mice]). ^#^*P* < .05, ^#^^#^*P* < .01, and ^#^^#^^#^*P* < .001, compared with the PBS or HDM group at ZT0 versus ZT12.

To determine whether pathophysiological changes were caused by acute HDM exposure at ZT0 and ZT12, the stress hormone corticosterone and serotonin levels in mice were measured. The serum corticosterone levels in female mice were significantly increased at ZT12 in the PBS and HDM groups compared with ZT0, and male mice showed a significant increase only at ZT12 in the HDM group compared with ZT0 (Fig 7). However, there was no significant change in serotonin levels in female and male mice at ZT0 and ZT12 in the HDM group compared with the respective PBS group (Fig 7). Interaction analysis revealed significant differences in serum corticosterone levels in the Time and Sex × Time response (Fig 7 and Table E1). We found no significant difference in corticosterone levels between PBS and HDM groups both at ZT0 and ZT12 in female and male mice combined from the acute HDM exposure. However, at ZT12, both PBS and HDM groups showed an increase in corticosterone levels compared with ZT0 in their respective PBS and HDM groups (see Fig E11, *A,* in this article’s Online Repository at www.jaci-global.org). Furthermore, there was no significant change in serotonin levels at ZT0 and ZT12 in the HDM group compared with their respective PBS group (Fig E11, *B*). Overall, we did not see any significant difference in stress hormone corticosterone levels in PBS versus HDM at ZT0 or ZT12.

### Acute HDM exposure augments lung inflammatory response in *Rev-erbα* KO mice

**Fig 8:**
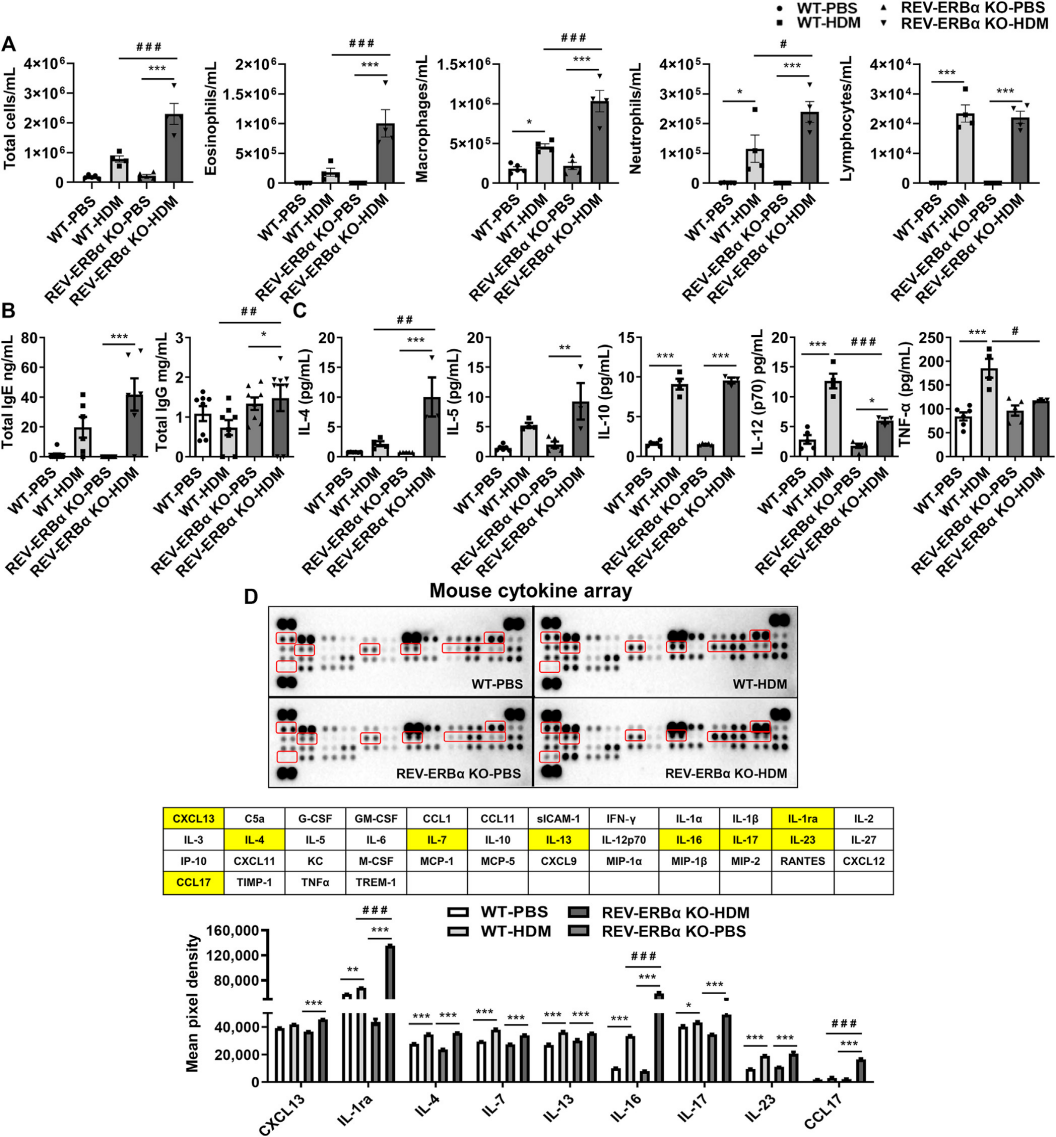
HDM challenge increases differential cell counts, immunoglobulin levels in plasma, and cytokines/chemokines in the BAL fluid of *Rev-erbα* KO mice. **A,** WT littermate and *Rev-erbα* KO mice were exposed to acute (10 days) PBS and HDM at ZT12 and euthanized 48 hours after the last exposure. Total cell counts in BAL fluid were determined by Acridine orange (AO) and propidium iodide (PI) staining using a Cellometer Auto 2000 (Nexcelom Bioscience, Lawrence, Mass). Total number of BAL macrophages, eosinophils, lymphocytes, and neutrophils were determined by differential cell counts performed using cytospin slides. **B,** Total IgE and IgG levels in serum were measured using ELISA. **C,** T_H_1/T_H_2 cytokines in BAL fluid were measured using a T_H_1/T_H_2 multiplex cytokine assay kit (Bio-Rad, Hercules, Calif). **D,** A mouse cytokine array was used to determine cytokine/chemokine profiles in pooled BAL fluid samples according to the manufacturer’s instructions (R&D Systems, Minneapolis, Minn). Cytokine array data marked in *red* and their corresponding order highlighted in *yellow* are presented as bar graphs with mean pixel density analyzed by ImageJ (version 1.54d; NIH, USA). Data are shown as mean ± SEM (n = 3-6/group). ^∗^*P* < .05, ^∗∗^*P* < .01, and ^∗∗∗^*P* < .001, significant compared with WT-PBS or *Rev-erbα* KO-PBS; ^#^*P* < .05, ^#^^#^*P* < .01, and ^#^^#^^#^*P* < .001, significant compared with WT-HDM.

HDM-exposed WT mice showed a significant increase in macrophages and neutrophils compared with PBS group. Lymphocytes were significantly increased in HDM-exposed WT and *Rev-erbα* KO mice compared with their respective PBS groups. HDM-exposed *Rev-erbα* KO mice showed significantly increased eosinophils, macrophages, and neutrophils compared with HDM-exposed WT mice (Fig 8, *A*). In addition, we found that HDM-exposed *Rev-erbα* KO mice produced higher serum levels of total IgE and IgG compared with PBS group (Fig 8, *B*). Multiplex cytokine analysis in BAL fluid of HDM-exposed *Rev-erbα* KO and WT mice revealed increased T_H_2 cytokines (IL-4 and IL-5) in HDM-exposed *Rev-erbα* KO mice compared with PBS group (Fig 8, *C*). IL-10 and IL-12 (p70) were significantly increased in HDM-exposed WT and *Rev-erbα* KO mice compared with the respective PBS group. However, TNF-α was increased in HDM-exposed WT mice but not in *Rev-erbα* KO mice (Fig 8, *C*). Complementary findings were observed when the pooled BAL fluid samples were analyzed in HDM-exposed WT and *Rev-erbα* KO mice compared with PBS control (significant increase in IL-4, IL-7, IL-13, IL-17, and IL-23 was observed in HDM-exposed WT and *Rev-erbα* KO mice compared with PBS group). Furthermore, HDM-exposed *Rev-erbα* KO mice showed augmented IL-1ra, IL-16, and CCL17 cytokine release compared with HDM-exposed WT mice, demonstrating REV-ERBα–dependent regulation of specific proinflammatory cytokines in the lungs (Fig 8, *D*).

### Acute HDM exposure differentially affects clock gene expression in isolated rEOSs and AMs

**Fig 9:**
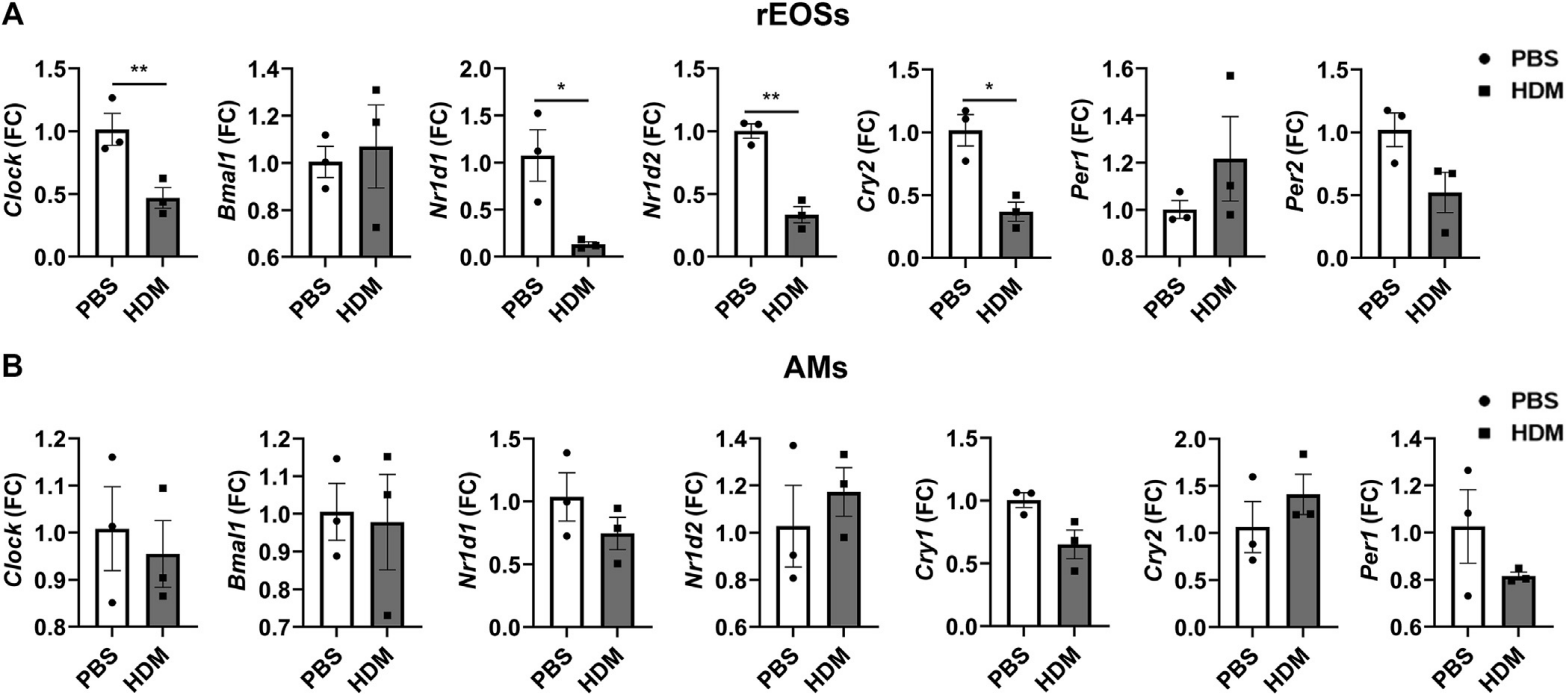
Acute HDM exposure affects circadian clock gene expression in immune cells. C57BL/6J female mice were exposed to acute (10 days) PBS and HDM at ZT6/12 PM and euthanized 48 hours after the last exposure at ZT6. Gene expression of core CCGs (*Clock*, *Bmal1*, *Nr1d1*, *Nr1d2*, *Cry1*, *Cry2*, *Per1*, and *Per2*) in fluorescence-activated cell–sorted (**A**) rEOSs and (**B**) AMs was determined (relative to 18S rRNA) by quantitative RT-PCR analysis and the results are presented in relation to expression changes in PBS group. Data are shown as mean ± SEM (n = 3/group). ∗*P* < .05 and ∗∗*P* < .01, compared with respective control (PBS) at ZT6.

Previous studies have shown the potential role of circadian clock genes in immune cells. Hence, we decided to determine the transcript levels of selected core clock genes in flow-sorted rEOSs that drive allergic airway inflammation along with AMs from PBS- and HDM-exposed WT mice. Interestingly, we found that acute HDM exposure caused significantly decreased expression of the core clock genes *Clock*, *Nr1d1*, *Nr1d2*, and *Cry2* in the isolated rEOSs but not in the AMs (Fig 9, *A* and *B*). However, the exact cell type–specific role of clock gene expression in immune cells during acute HDM-induced allergic asthma remains unclear.

### Acute HDM exposure affects the locomotor activity of mice

In a separate experiment, we investigated the effect of acute HDM exposure on the circadian rhythm of wheel-running behavior in WT mice. The wheel-running behavior of mice was measured to determine whether acute HDM exposure causes a reduction in overall wheel-running activity. Acute HDM-exposed mice showed a significant reduction in the nocturnal activity (active phase/dark cycle) from day 5 post–initial HDM exposure until day 10 (see Fig E12, *A* and *B***,** in this article’s Online Repository at www.jaci-global.org). Analysis of total ambulatory counts across the 24-hour day revealed that acute HDM exposure significantly reduced their activity 5 days post–HDM exposure. Average ambulatory counts pre-exposure and during the course of HDM exposure revealed a significant reduction in allergen-induced wheel-running behavior during the dark cycle/active phase compared with PBS exposure (Fig E12, *B*). These findings support that acute HDM exposure significantly affects the behavioral rhythms of mice, which may have additionally contributed to the observed difference in outcomes and phenotypes reported in the study.

## Discussion

HDM is the most common human allergen in urban areas, and its exposure results in increased airway hyperresponsiveness and remodeling via an exaggerated immune-inflammatory response in the lungs. Nocturnal worsening of symptoms/severity in asthma support the involvement of circadian rhythms in chronic airway disease. Previous studies have shown variation in asthma response depending on the time of HDM exposure.^12^^,^^15^^,^^16^ Additional studies have reported diurnal variation in lung mechanics and altered immune-inflammatory cell homing in the lung and its effects during time-of-day allergen challenges.^17^^,^^18^ For the first time, we show a time-of-day variation in lung immune cell homing and humoral response following acute HDM exposure in mice. HDM challenge at ZT12 in mice (dark cycle: active phase), which is equivalent to morning (dawn) in humans, caused an exaggerated myeloid cell homing in the lungs compared with ZT0 (light cycle: resting phase). It is worth reiterating that these significant changes were observed in the acute HDM exposure model 48 hours after 10 days of exposure.

To date, there are no studies that demonstrate a time-of-day and sex-based difference in response to HDM in a preclinical mouse model of asthma. Our immunophenotyping analysis showed that eosinophil (iEOS, rEOS, and GR1^+^ EOS) and macrophage (AM and IM) influx into the airway in response to acute HDM exposure was mostly affected at ZT12 compared with ZT0. Other inflammatory cells, such as neutrophils and DCs, did not show a time-of-day response to acute HDM exposure. HDM-exposed female and male mice also showed a time-of-day difference in the degree of lung inflammation (heightened immune response in female versus male mice at ZT12) associated with altered circadian clock gene expression. Overall, HDM-exposed female and male mice at ZT12 showed augmented inflammatory response in the lung associated with asthmatic phenotypes including inflammatory chemokines/cytokines, airway inflammation, and mucus production.

The myeloid cell infiltration in BAL fluid and lung tissue of female mice complement the findings, but male mice showed some differences in their outcomes such as no time-of-day response in eosinophil subsets in lung tissues. The BAL fluid immunophenotyping may be more reflective of the entire lung, whereas only the larger left lobe was used for the analysis conducted using lung tissues. A recent study showed a time-of-day difference in airway hyperresponsiveness and lung mechanics following acute HDM exposure at ZT0 versus ZT12 but reported no difference in immune cell infiltration in the lung (BAL fluid). The lack of thorough investigation of myeloid cell subsets from the previous study may have resulted in this conflicting result.^18^ In addition, other factors such as duration and dose of HDM (10 days [30 μg HDM; equal number of female and male mice] versus 15 days [5 d/wk for 3 weeks; 25 μg HDM; female WT mice]) may have contributed to the difference observed between the previous report and this study. Ongoing studies in our laboratory will address whether chronic HDM exposure (∼5 weeks) shows exaggerated immune response including other immune subsets such as lymphocytes and innate lymphoid cells that may be different compared with what is observed in the acute (10 days) HDM exposure model. Total IgE levels showed a time-of-day response to HDM exposure at ZT12, and female mice showed higher IgE response than the male mice at ZT12. However, total IgG remained unaffected by acute HDM exposure at ZT0 and ZT12. Unlike total IgG, HDM-specific IgG and its subtypes were elevated in the HDM group without showing time-of-day response. Importantly, our results suggest that allergic IgE-mediated responses appear to be under a greater influence of the circadian clock.^19^

Corticosterone is a stress hormone known for its anti-inflammatory and immunosuppressive response, and its diurnal oscillation is tightly regulated by the circadian clock.^20^ We measured serum corticosterone levels in PBS- and HDM-exposed mice at ZT0 and ZT12. Our findings suggest that HDM exposure does not affect corticosterone levels in mice. As expected, there was a time-of-day response observed at ZT0 versus ZT12 in both the PBS and HDM groups (increased corticosterone levels at the beginning of the active phase ZT12).^21^ Corticosterone is well known for its rhythmic change in serum levels during the light and dark cycles. It is believed to be an important entraining cue for the synchronization of the peripheral clocks to the central clock located in the suprachiasmatic nucleus of the brain.^22^ Our previous study showed that cigarette smoke exposure–induced lung inflammation caused an abnormal phase shift in the rhythms of plasma corticosterone and serotonin levels in mice.^23^ However, we found that time-of-day response in serum corticosterone levels increased significantly at ZT12 independent of PBS and HDM exposure, suggesting that acute HDM-induced circadian clock disruption in the lung did not affect stress hormone levels systemically in this model.

Cytokines/chemokines secreted by airway epithelial cells during HDM exposure regulate other key processes such as the accumulation and activation of different myeloid cell types in the lung. NanoString analysis of myeloid innate immunity panel corroborates with lung inflammation at ZT12 in the HDM group, showing increased expression of chemokines *Ccl2, Ccl8, Ccl9, Ccl12, Cxcl1, Cxcl5*, and *Cxcl10*. Chemokines such as CCL2 and CCL8 are involved in the recruitment of eosinophils into the lung.^24^ In addition, CCL12 is implicated in eosinophil egress from bone marrow in response to allergen exposure by binding to CXCR4.^25^ The time-of-day response in eosinophil accumulation observed in the lung may be through rhythmic variation in chemokine expression (*Ccl2, Ccl8*, and *Ccl12*). However, chemokine gene *Ccl9* showed a time-of-day response at ZT0 compared with ZT12 in the HDM group. The previous study supports that allergen-induced airway inflammation caused an accumulation of eosinophils, which increases the expression and release of chemokines in the lungs.^26^ Interestingly, acute HDM exposure did not show a significant difference in neutrophil infiltration; conversely, neutrophil-specific chemokine expression in the lung (*Cxcl1* and *Cxcl5*) showed a time-of-day gating response at ZT0 compared with ZT12. We found that *Cxcl10* mRNA was significantly upregulated at ZT12 in the HDM group compared with the PBS group, which may have a profound role in the chemotaxis of immune cells including eosinophils, monocytes, and T lymphocytes shown previously.^27^

Neutralizing antibodies/biologics–based therapies are gaining popularity in the treatment of asthma.^28^ Targeting chemokine/cytokine receptors could be more effective because they are expressed by multiple immune cells and may be more effective in controlling inflammation and associated lung phenotypes.^29^ Transcriptomic analysis revealed multiple chemokine/cytokine receptors (*Cxcr5, Ccr2, Tnfrsf1b*, and *Pdcd1lg2)* that were highly expressed at ZT12 in the HDM group. CCR2 receptor regulates eosinophil and macrophage infiltration, and CXCR5 plays an important role during antibody maturation and class switching, which may be a key target for asthma therapy.^30^^,^^31^ Studies to mechanistically understand biologics that target CCR2 and CXCR5 along with chronotherapeutic approaches will provide a new direction in the treatment of asthma. In addition, we found that transcript levels of *Stat5b, Tnfrsf12a*, and *Sqstm1* were significantly downregulated at ZT12 in the HDM group, which are shown to be involved in inflammation and airway remodeling.^32^, ^33^, ^34^ Furthermore, other novel candidate genes upregulated at ZT12 in the HDM group that showed time-of-day gating responses including *Fpr-rs4, Arg1, Lat2, Adamts3*, and *Adam8* are involved in T-cell activation, immune cell metabolism, mast cell activation, and extracellular matrix (ECM) remodeling. NanoString data comprehensively provide deeper insights into the time-of-day gating response observed in myeloid immunity target genes following acute HDM exposure.

HDM exposure also showed a differential response in the gene expression of core circadian clock-controlled genes (CCGs) in female and male mice. Most of the core CCGs were significantly altered at ZT12 in the HDM group compared with ZT0. Altered circadian clock genes at ZT12 in the HDM group may correlate and explain the variation in immune response at ZT12 (immigration of immune cells into peripheral organs at the onset of the behavioral active phase).^8^ These findings support that circadian clock genes are highly sensitive to environmental changes or perturbations during the active phase (dark cycle/night) compared with the resting phase (light cycle/day). HDM-induced airway inflammation is associated with altered mRNA levels of core CCGs (*Per1*, *Per2*, *Per3*, *Nr1d1*, *Nr1d2*, and *Dbp*) in the lungs of female and male mice compared with respective PBS control at ZT12*.* Similar findings were reported for *Bmal1* and *Rev-erbα/Nr1d1* gene expression in 15-day HDM-exposed mouse lungs compared with PBS control.^18^ However, the exact reason for altered gene expression of CCGs in the lung and sex-based differences following acute HDM exposure needs further investigation. Our findings demonstrate that female mice show higher circadian disruption (reduced expression of core clock genes) at ZT12 than male mice. Similarly, another human study showed sex-based differences in circadian alterations pronounced in women compared with men in the early morning on the basis of monitoring their cognitive functions after forced desynchrony.^35^ Overall, our data highlight the importance of differentiating female and male mice in preclinical studies to demarcate time-of-day response and sex-based differences in HDM-induced immune-inflammatory response and asthmatic lung phenotypes that imply other chronic airway diseases.

*Rev-erbα* KO mice exposed to 10 days of HDM showed exaggerated inflammation associated with increased total cells, eosinophils, macrophages, and neutrophils compared with HDM-exposed WT mice. In addition, HDM-exposed *Rev-erbα* KO mice showed increased total IgE and IgG levels and T_H_2 cytokine release in serum and BAL fluid, respectively. These findings support that circadian clock disruption augments HDM-induced allergic airway inflammation in mice. Cytokine array data support the observed heightened inflammatory response and REV-ERBα regulated cytokines in the lung following acute HDM exposure. Our findings are in line with another study that showed that 15 days of HDM exposure caused increased time-of-day effects on airway hyperresponsiveness in WT mice (maximal at the active phase/dark cycle) but not in *Rev-erbα* KO mice, suggesting a potential involvement of muscarinic receptors (*Chrm1* and *Chrm3*).^18^ However, the authors did not conduct a systematic evaluation of time-of-day response in lymphoid and myeloid cells (BAL fluid and lung tissues) and/or other parameters including the sex-based differences in the acute HDM exposure model. We for the first time showed that several core circadian clock genes were significantly downregulated in isolated rEOSs in acute HDM-exposed mice, suggesting a possible role of the circadian clock in immune cells during asthma. In a separate experiment, we also determined the locomotor activity of mice pre– and post–HDM exposure to determine the effect of acute allergen exposure on wheel-running behavior. We observed a significant reduction in nocturnal locomotor activity in mice 5 days post–HDM exposure until day 10. Overall, these findings are interesting and support the potential role of circadian clock disruption associated with the time-of-day response and sex-based differences in airway inflammation *in vivo* in acute HDM-induced allergic asthma.

This study has a few limitations. We focused our work using the most commonly used intranasal route of HDM exposure in female and male WT mice. We believe the route of exposure can potentially have an impact on the overall outcome analyzed. We conducted only 1 experiment using WT and *Rev-erbα* KO mice (combined female and male mice) at the ZT12 time point following acute HDM exposure because of the limited availability of male and female *Rev-erbα* KO mice. In addition, we have not analyzed the changes in lung lymphocyte (CD4^+^ and CD8^+^) and innate lymphoid cell subsets as part of the study because we mainly focused on myeloid cells in BAL fluid and lung tissues. The observed sex-based and diurnal differences in acute HDM exposure cannot be generalized to other experimental models of allergic asthma unless and until tested appropriately, which is beyond the scope of the present study. We are currently conducting studies to address whether a similar time-of-day response and the sex-based differences are observed using another allergen (*Alternaria alternata*) or with a different HDM administration protocol (other acute and chronic allergen sensitization and challenge models). Future studies will provide evidence to support the relevant findings on time-of-day response, sex-based differences, and the role of circadian clock disruption in allergic asthma *in vivo* in mice.

For the first time, we showed that acute HDM exposure exhibits sex-based differences and time-of-day exaggerated response at ZT12 associated with circadian clock disruption (reduced expression of clock genes) in the lung. HDM-exposed female mice showed a strong time-of-day response at ZT12 with increased myeloid cell infiltration that correlates with IgE-mediated response. This study demonstrates the importance of conducting preclinical studies using an equal number of female and male mice, and analyzing the data together and separately to clearly distinguish the phenotypes that may show a sex-based difference *in vivo*. Our study revealed that female mice exhibit a stronger time-of-day difference in the altered expression of core CCGs in the lungs. However, analyzing the status of clock genes in the lung structural cells and specific immune cells will be essential to delineate the exact molecular mechanism of circadian clock disruption and altered time-of-day response following HDM exposure. Future studies will shed light on the role of core clock genes that contribute to the overall allergic response in asthma.

## Disclosure statement

This work was supported in part by the National Institutes of Health (NIH), National Heart, Lung, and Blood Institute (grant no. R01HL142543 to I.K.S.), the 10.13039/100000057National Institute of General Medical Sciences (grant no. P20 GM103418), as well as the 10.13039/100006727University of Kansas Medical Center, 10.13039/100008235School of Medicine, Internal Medicine Start-Up Funds (I.K.S.).

Disclosure of potential conflict of interest: The authors declare that they have no relevant conflicts of interest.

## Supplementary data

Appendix E1

Supplementary Tables E1-E3

Supplementary Figures E1-E12
