# Supplementary material for: Acute HDM exposure shows time-of-day and sex-based differences in the severity of lung inflammation and circadian clock disruption
Source: J Allergy Clin Immunol Glob. 2023 Jul 24;2(4):100155. doi: 10.1016/j.jacig.2023.100155 (PMC10509939; doi:10.1016/j.jacig.2023.100155)
Supplement: Appendix E1 [file mmc1.docx]

**Online Supplement Appendix E**

**Title: Acute HDM exposure shows time-of-day and sex-based differences in the severity of lung inflammation and circadian clock disruption**

**Authors:** Ashokkumar Srinivasan ^#^, Allan Giri, Santhosh Kumar Duraisamy, Alexander Alsup, Mario Castro, and Isaac Kirubakaran Sundar ^# *^

Division of Pulmonary, Critical Care and Sleep Medicine, Department of Internal Medicine, University of Kansas Medical Center, Kansas City, KS, USA

^#^ Authors contributed equally to this study.

**METHODS**

***Experiment Animals***

8-week-old wild-type C5BL/6J male and female mice, weighing approximately 20 to 24 grams, were purchased from the Jackson Laboratory (Jax mice, Bar Harbor, ME). In another experiment, we utilized WT littermates and *Rev-erbα* KO mice (~2-3 months old; female and male) obtained from the Jacson Laboratory (Strain # 018447; https://www.jax.org/strain/018447). All animals were housed at the University of Kansas Medical Center (KUMC) vivarium in a controlled environment with a 12:12-h light-dark cycle and *ab libitum* access to food and water. All experiments were performed in agreement with the Institutional Animal Care and Use Committee (IACUC) of KUMC (Protocol Number: 2020-2575) and the National Institute of Health (NIH) Guide for Care and Use of Laboratory Animals.

***Bronchoalveolar lavage (BAL) fluid***

Mice were injected with Phenytoin/Pentobarbital (50 mg/kg body weight; Euthanasia solution, Paterson Veterinary Supply, Inc. MA, USA) via intraperitoneal route and euthanized by exsanguination. The lungs were lavaged with 0.6 ml of sterile saline (0.9% sodium chloride) as described previously ^E1^. Lavage fluid-containing cells were centrifuged and resuspended in 1 ml of BD staining buffer for total cell counting using Countess 3 Automated Cell Counter (Thermo Fisher Scientific, Waltham, MA, USA). In brief, 10 µl of cell suspension was mixed with 10 µl of trypan blue viability dye and counted using the Countess cell counting chamber slide. BAL fluid (cell-free supernatant) was stored in 2 ml tubes at -80 ºC for proinflammatory cytokine/chemokine analysis.

In another experiment using WT littermates and *Rev-erbα* KO mice, the BAL fluid was collected from the lungs by lavage (3 times using 0.6 ml saline) via a cannula inserted into the trachea as described previously ^E1^. The BAL fluid was centrifuged at 2000 rpm and the cell-free supernatants were stored at -80 °C for cytokine analysis. The inflammatory cell pellets were resuspended in 1 ml saline to determine the total cell counts using cellometer. Then, cytospins were prepared at 50,000 cells/slide in duplicates, and differential cell counts were performed in cytospin slides with Diff Quik (Dade Behring, Newark, DE, USA). Th1/2 cytokines in BAL fluid were measured using the Th1/2 multiplex cytokine assay kit (Bio-Rad Laboratories). The results were expressed as picograms per milliliter for cytokines measured in BAL fluid. Similarly, pooled BAL fluid (n=5/group) was used to determine cytokine/chemokine using a mouse cytokine array according to the manufacturer’s instructions (R&D systems).

***Lung Digestion***

Lung tissue (larger left lobe) was cut into small pieces and resuspended in 5 ml digestion buffer [DMEM F12 media containing Liberase (2.6 IU) and DNase I (30 µg)]. Tissue digestion was carried out in a shaking incubator at 37 ˚C for 1 h. After incubation, single-cell suspension was made by repeated pipetting of the dissociated cells followed by sequential filtering through a 70-micron and 40-micron nylon mesh to remove clumped cells and undigested tissues. Dissociated lung cells were washed and resuspended in BD stain buffer (554656; BD Bioscience). Total cells in dissociated lung tissues were counted using the Countess 3 Automated cell counter as described previously. Dissociated lung cells were immediately stained using specific antibodies for flow cytometry analysis.

***Flow cytometer analysis***

Dissociated lung cells (1x10^6^ in 100 µL) were resuspended in staining buffer and blocked with anti-CD16/32 antibody (101302; BioLegend San Diego CA, USA) and subsequently stained using the following antibodies: anti-CD45 BV605 (103155; BioLegend), anti-CD3 PE-Cy7 (100320; BioLegend), anti-CD11b (101226; BioLegend), anti-IA/IE BV650 (107641; BioLegend), anti-CD11c (117336; BioLegend), anti-CD24 AF488 (101816 BioLegend), anti-CD193 PE (144506; BioLegend), anti-Gr1 AF700 (127622; BioLegend), anti-CD64 APC (139306; BioLegend), anti-Siglec-F (562757; BD Bioscience), and Ghost dye 510 (Tonbo bioscience) for live and dead stain to remove dead cells from the analysis. The list of antibodies, catalog number, and company name(s) used for immunophenotyping analysis in this study are summarized (**Table E2**). The data acquisition was performed with LSRII fortessa by collecting 100,000 cells/sample and analyzed using FCS Express 7 software. Compensation for each flow cytometer experiment was performed with unstained and all single-color controls using OneComp eBeads (ThermoFisher Scientific). Representative dot plots of a multicolor flow cytometry panel were used to identify myeloid cell subsets in BAL fluid and lung tissues from mice were provided as described previously (**Fig E2A-B**) ^E2^. The sex-based difference in myeloid cells from BAL fluid and lung tissues was analyzed using the combined dataset presented in the same study. In a separate experiment, C57BL/6J female mice were exposed to acute (10d) PBS and HDM at ZT6 (12:00 pm). Lung resident eosinophils and alveolar macrophages were sorted using BD FACS Aria Illu (50,000 to 100,000 cells from each sample) and stored in Trizol for downstream processing.

***Total IgE and IgG ELISA***

The total serum IgE and IgG levels were measured using commercially available ELISA kits (Bethyl Laboratories, Inc. TX, US) according to the manufacturer’s protocol. The serum sample was diluted at 1:5 and 1:25,000 for total IgE and IgG, respectively. In brief, 96 well ELISA plate was coated with anti-IgE/IgG capture antibody and blocked with 5% bovine serum albumin for 2 h, followed by the addition of serum samples in duplicates with the above-mentioned dilution in respective wells. After 1 h incubation, the ELISA plates were washed three times with 1X PBS containing 0.05% Tween 20. Then, the biotin-conjugated detection antibody was added and incubated at room temperature (RT) for 1 hr. After three washes using the wash buffer, the Streptavidin-HRP was added and incubated for 30 min. After the final washes, 3,3',5,5'-Tetramethylbenzidine (TMB) substrate was added and incubated for 30 min and the color reaction was stopped using 2N sulfuric acid. Using the endpoint kinetic method, the intensity of color development was measured at 450 nm which directly correlates with standard concentration. The data were expressed as ng/ml for total IgE and mg/ml for total IgG.

***HDM-specific Immunoglobulins ELISA***

HDM-specific immunoglobulins (IgE, IgA, IgM, IgG, IgG_1_, IgG2b) in serum were measured using commercially available ELISA kits (Chondrex, Inc. WA USA) according to the manufacturer’s protocol. The serum sample was diluted at 1:10 for HDM-specific immunoglobulins (IgA, IgM, and IgG2b) and 1:100 for HDM-specific IgG and IgG1. HDM-specific IgE was measured using a 96-well ELISA plate coated with anti-mouse IgE followed by detection of HDM-specific IgE using biotinylated HDM and the result was expressed as absorbance at 450 nm. For all the remaining HDM-specific immunoglobulins, HDM coated 96 well ELISA plate was used to measure using standard sandwich ELISA according to the manufacturer’s protocol.

***Corticosterone and Serotonin ELISA***

Corticosterone and serotonin levels in the serum of acute HDM exposed mice were analyzed using commercially available ELISA kits (Enzo Life Sciences, Inc. NY, USA,) according to the manufacturer’s protocol. The serum sample was diluted to 1:40 and 1:32 for corticosterone and serotonin, respectively. In brief, corticosterone and serotonin were measured using a competitive immunoassay with alkaline phosphatase and p-Nitrophenyl Phosphate (PNPP) as the detection system. The color reaction was stopped using trisodium phosphate solution. The optical density of samples was measured at 405 nm, which inversely correlates with the absorbance of standards.

***Histological Analysis and Immunohistochemical Staining***

Intact lung lobes were inflation-fixed with 1% low melting agarose in PBS and immersion in 10% neutral buffered formalin (NBF) solution (Sigma-Aldrich). After 48 hours post-fixation in NBF solution, the lung lobes were prepared for paraffin embedding by progressive dehydration using 30%, 50% and 70% Ethanol. Lung lobes were carefully identified, paraffin-embedded, and cut into 5-μm sections for various histochemical staining followed by analyses.

***Hematoxylin and Eosin (H&E) staining***

H&E staining was performed to semi-quantitatively assess the degree of inflammation in peribronchial (airways), vascular/perivascular and alveolar regions of the lung sections. A five-point grading system (0 – 4) was used to manually and blindly score the lung sections, where 0 indicated normal lung with almost no infiltration of immune cells, 1 indicated slight inflammation with a few cells scattered around the area of interest but did not create any depth, 2 indicated mild inflammation with a few layers of cells deep surrounding the area of interest but not so dense that it would surround other blood vessels, 3 indicated moderated inflammation with four to five layers of cells surrounding the area of interest and often dense enough to border the surrounding structures, and 4 indicated severe inflammation with almost the entire area of interest surrounded with more than four to five layers of cells and always engulfing the surrounding structures. When it was difficult to decide between two grades, the average of the two grades was given. Finally, the mean inflammation score was obtained for each lung section in the control (PBS) and HDM exposed groups for statistical analysis.

***Periodic-Acid-Schiff (PAS) Staining***

PAS staining was performed to assess mucin production in larger airways of the lung. PAS staining kit was obtained from Sigma-Aldrich (SKU: 395B-1KT) and lung sections were stained using the manufacturer’s protocol. A five-point grading system (0 – 4) was used to manually and blindly score the lung sections, where 0 indicated less than 0.5% of PAS-positive cells with almost no staining of the airway epithelium (normal lung), and 1, 2, 3 and 4 indicated less than 25%, 25 to 50%, 50 to 75% and more than 75% of the airway epithelium staining respectively. When it was difficult to decide between two grades, the average of the two grades was given. Finally, the mean score was obtained for each lung section in the control (PBS) and HDM exposed groups for statistical analysis. It is important to note that the final score used for the statistical analysis is reflective of the overall PAS-positive staining in sections of the same lung lobe that was scored.

***Masson’s Trichrome***

Trichrome staining was performed to visualize and semi-quantitatively analyze the degree of collagen deposition surrounding peribronchial regions. Trichrome staining kit was obtained from Sigma-Aldrich (SKU HT15-1KT) and lung sections were stained using the manufacturer’s protocol. A five-point grading system (0 – 4) was used to manually and blindly score the lung sections, where 0 indicated normal lung, 1 indicated minimal collagen deposition but no observable thickening surrounding the bronchial vessel, 2 indicated mild thickening of the peribronchiolar region and just enough that it created an observable depth, 3 indicated moderate thickening around the peribronchial region and dense enough that it surrounded nearby blood vessels, and 4 indicated severe thickening of the peribronchial region and to an extent that distorted the surrounding structures. When it was difficult to decide between two grades, the average of the two grades was given. Finally, the mean score was obtained for each lung section and used for statistical analysis.

***Nanostring mRNA profiling***

Total RNA samples were diluted to 100 ng RNA concentration for NanoString nCounter SPRINT profiler using the Mouse Myeloid Innate Immunity v2 panel (NanoString Technologies, Inc. Cat# XT-CSO-MMII2–12). Raw counts were normalized using three housekeeping genes (*Eef1g, Oaz1, and Tubb5*) with higher average counts and lower %CV for normalization to obtain normalized counts. After normalization, transcripts with more than 100 counts were considered for the data analysis, and normalized raw counts were used for comparison between PBS and HDM groups at ZT0 and ZT12 using the nSolver analysis software (version 4.0) ^E3^. Selected target genes identified in mouse myeloid innate immunity panel were previously reported as part of the miRNA-mRNA target validation in our recent study ^E3^.

***Gene expression by quantitative real-time PCR (qRT-PCR)***

Lung tissues harvested from acute HDM exposed mice and PBS control were flash-frozen in liquid nitrogen and stored at -80 °C. Lung tissues (~50 mg) were homogenized in Trizol and total RNA was isolated using the RNeasy mini kit (Qiagen) according to the manufacturer’s instructions. Similarly, in a separate experiment, we utilized flow sorted resident EOS and AMs from PBS and HDM exposed WT mice to isolate total RNA. Total RNA quality and quantity were measured using a Nanodrop spectrophotometer (Nanodrop One; Thermo Scientific) followed by cDNA synthesis (1 μg of total RNA) with the First Strand cDNA synthesis kit (Qiagen). Prepared cDNA was stored at -20°C and used for qRT-PCR analysis using CFX 96 Real-Time PCR system (Bio-Rad). Either PrimePCR SYBR Green Assay primers (Bio-Rad) for mouse circadian clock genes (*Clock*: qMmuCED0046959, *Bmal1* [*Arntl*]: qMmuCED0049609, *Per1*: qMmuCED0045068, *Per2*: qMmuCID0020035, *Per3*: qMmuCID0021918, *Cry1*: qMmuCID0012363, *Cry2*: qMmuCID0019665, *Nr1d1* [*Rev-erbα*]: qMmuCID0014284, *Nr1d2* [*Rev-erbβ*]: qMmuCED0045882, *Nfil3*: qMmuCID0020254, *Dbp*: qMmuCID0009975, *Gm129*: qMmuCED0025109, *Hlf*: qMmuCED0044952, *Rora*: qMmuCED0061018, *Rorc*: qMmuCID0006484, *Bhlhe40*: qMmuCED0040322, and *Bhlhe41*: qMmuCED0047592) or custom made primers from Integrated DNA Technologies (IDT) were used (**Table E2**). For normalization, 18S ribosomal RNA (*Rn18s*) was used which is considered a stable housekeeping control ^E4^. The Ct value for *Rn18s* was subtracted from the Ct value for the gene of interest to obtain a delta Ct (ΔCt) value. The relative fold-change for each target gene was calculated using the 2^-ΔΔCt^ method as described previously ^E4, E5^. The list of gene-specific qRT-PCR primers used in the study is provided (**Table E3**).

***Mouse wheel-running activity (Locomotor activity)***

Wild-type male mice (Balb/c) were individually housed in standard mouse cages fitted with a stainless-steel running wheel (Coulbourn Instruments, Flushing, NY, USA) placed within a light-tight chamber (Phenome Technologies, Lincolnshire, IL, USA). Wheel-running activity was recorded in 1 min intervals and analyzed with ClockLab software (Actimetrics, Evanston, IL, USA). The experiments were performed in a regular 12:12 L:D cycle throughout the period (6 days pre-exposure [before] and 10 days [during PBS or HDM exposure].

**References:**

E1. Sundar IK, Rashid K, Gerloff J, Rangel-Moreno J, Li D, Rahman I. Genetic ablation of histone deacetylase 2 leads to lung cellular senescence and lymphoid follicle formation in COPD/emphysema. FASEB J 2018; 32:4955-71.

E2. Yu YR, O'Koren EG, Hotten DF, Kan MJ, Kopin D, Nelson ER, et al. A Protocol for the Comprehensive Flow Cytometric Analysis of Immune Cells in Normal and Inflamed Murine Non-Lymphoid Tissues. PLoS One 2016; 11:e0150606.

E3. Sundar IK, Srinivasan A. Lung miRNA profiles show a time-of-day response in house dust mite-induced allergic asthma in mice. Clin Transl Allergy 2021; 11:e12057.

E4. Giri A, Sundar IK. Evaluation of stable reference genes for qPCR normalization in circadian studies related to lung inflammation and injury in mouse model. Scientific Reports 2022; 12.

E5. Sundar IK, Rahman I. Gene expression profiling of epigenetic chromatin modification enzymes and histone marks by cigarette smoke: implications for COPD and lung cancer. Am J Physiol Lung Cell Mol Physiol 2016; 311:L1245-L58.
