# Supplementary material for: Acute HDM exposure shows time-of-day and sex-based differences in the severity of lung inflammation and circadian clock disruption
Source: J Allergy Clin Immunol Glob. 2023 Jul 24;2(4):100155. doi: 10.1016/j.jacig.2023.100155 (PMC10509939; doi:10.1016/j.jacig.2023.100155)
Supplement: Supplementary Tables E1-E3 [file mmc2.doc]

**Supplemental Tables:**

**Table E1 Interaction analysis (p-value) for Treatment, Sex, and Time responses (Two-way and Three-way interactions) presented in this study.**

| **Fig. 1** | **Total Cells** | **iEOS** | **GR1+EOS** | **AV MACs** | **iMACs** | **Neutrophils** | **DC** |  |  |  |
| --- | --- | --- | --- | --- | --- | --- | --- | --- | --- | --- |
| # of samples/group | 4-7 | 4-7 | 4-7 | 4-7 | 4-7 | 4-7 | 4-7 |  |  |  |
| Treatment | 0.0018384 | 0.0569045 | 0.0795685 | 0.0265533 | 0.0284099 | 0.0526322 | 0.0313492 |  |  |  |
| Sex | 0.1009682 | 0.1701421 | 0.0846191 | 0.8061128 | 0.4092392 | 0.3485202 | 0.033606 |  |  |  |
| Time | 0.0203503 | 0.0154294 | 0.0329272 | 0.6294897 | 0.0004649 | 0.0543159 | 0.0452838 |  |  |  |
| Treatment x Sex | 0.3193316 | 0.3768606 | 0.2659966 | 0.8354932 | 0.5957585 | 0.5469472 | 0.1640684 |  |  |  |
| Treatment x Time | 0.1227365 | 0.0979884 | 0.1467965 | 0.1864319 | 0.0214584 | 0.1939966 | 0.1696946 |  |  |  |
| Sex x Time | 0.6797582 | 0.083947 | 0.3812512 | 0.1831456 | 0.1025666 | 0.2962305 | 0.1707247 |  |  |  |
| Treatment x Sex x Time | 0.7967729 | 0.2373162 | 0.5465679 | 0.3695285 | 0.2824063 | 0.4772103 | 0.3481866 |  |  |  |
|  |  |  |  |  |  |  |  |  |  |  |
| **Fig. 2** | **rEOS** | **AV MACs** | **iMACs** | **Neutrophils** | **DC** | **T cells (CD3+)** |  |  |  |  |
| # of samples/group | 5-7 | 5-7 | 5-7 | 5-7 | 5-7 | 5-7 |  |  |  |  |
| Treatment | 0.0002648 | 0.01 | 0.0011842 | 0.0739121 | 0.8012679 | 0.7076965 |  |  |  |  |
| Sex | 0.7361815 | 0.0287579 | 0.6344289 | 0.3432316 | 0.0072294 | 0.6334318 |  |  |  |  |
| Time | 0.0000011 | 0.012592 | 0.9271922 | 0.4707981 | 0.0199905 | 0.0502599 |  |  |  |  |
| Treatment x Sex | 0.9463197 | 0.016514 | 0.7984587 | 0.9952412 | 0.9979076 | 0.4816307 |  |  |  |  |
| Treatment x Time | 0.0001882 | 0.0072934 | 0.9530389 | 0.720783 | 0.2079243 | 0.1289635 |  |  |  |  |
| Sex x Time | 0.0001687 | 0.1342161 | 0.0006448 | 0.5732159 | 0.1413388 | 0.0027789 |  |  |  |  |
| Treatment x Sex x Time | 0.0068421 | 0.4430151 | 0.09551 | 0.9484821 | 0.0070817 | 0.1770136 |  |  |  |  |
|  |  |  |  |  |  |  |  |  |  |  |
| **Fig. 3** | **Total IgE** | **Total IgG** | **HDM-IgE** |  |  |  |  |  |  |  |
| # of samples/group | 5-6 | 5-6 | 6-13 |  |  |  |  |  |  |  |
| Treatment | 0.0346342 | 0.3146655 | 0.5015376 |  |  |  |  |  |  |  |
| Sex | 0.064742 | 0.1838559 | 0.0015994 |  |  |  |  |  |  |  |
| Time | 0.0000236 | 0.2803994 | 1 |  |  |  |  |  |  |  |
| Treatment x Sex | 0.1825834 | 0.9248617 | 0.2160774 |  |  |  |  |  |  |  |
| Treatment x Time | 0.001111 | 0.3492572 | 0.417176 |  |  |  |  |  |  |  |
| Sex x Time | 0.0656285 | 0.8640472 | 0.0904993 |  |  |  |  |  |  |  |
| Treatment x Sex x Time | 0.2315575 | 0.4291304 | 1 |  |  |  |  |  |  |  |
|  |  |  |  |  |  |  |  |  |  |  |
|  |  |  |  |  |  |  |  |  |  |  |
|  |  |  |  |  |  |  |  |  |  |  |
| **Fig. 4** | **HDM-IgG** | **HDM-IgG1** | **HDM-IgG2b** | **HDM-IgG3** | **HDM-IgA** | **HDM-IgM** |  |  |  |  |
| # of samples/group | 5 | 5 | 5 | 5 | 5 | 5 |  |  |  |  |
| Treatment | 0.0037114 | 0.0485902 | 0.0013 | 0.1699977 | 0.0979146 | 0.0858693 |  |  |  |  |
| Sex | 0.9215784 | 0.2205551 | 0.25396 | 0.784627 | 0.2497143 | 0.6433233 |  |  |  |  |
| Time | 0.6102771 | 0.9842324 | 0.9557738 | 0.072017 | 0.7270664 | 0.8300294 |  |  |  |  |
| Treatment x Sex | 0.1754158 | 0.8720478 | 0.1683123 | 0.2657425 | 0.0282365 | 0.3426185 |  |  |  |  |
| Treatment x Time | 0.3355924 | 0.899304 | 0.3444605 | 0.4744795 | 0.3549025 | 0.3669534 |  |  |  |  |
| Sex x Time | 0.3473333 | 0.8380643 | 0.1961721 | 0.2864027 | 0.4377574 | 0.8264819 |  |  |  |  |
| Treatment x Sex x Time | 0.7581351 | 0.5276339 | 0.9552773 | 0.6476421 | 0.8091689 | 0.3301922 |  |  |  |  |
|  |  |  |  |  |  |  |  |  |  |  |
| **Fig. 6** | **Clock** | **Bmal1** | **Per1** | **Per2** | **Per3** | **Cry1** | **Cry2** | **Nr1d1** | **Nd1d2** | **Dbp** |
| # of samples/group | 4-5 | 4-5 | 4-5 | 4-5 | 4-5 | 4-5 | 4-5 | 4-5 | 4-5 | 4-5 |
| Treatment | 0.2804851 | 0.1294767 | 0.616009 | 0.9506152 | 0.9471933 | 0.5968158 | 0.6794986 | 0.84367 | 0.9096596 | 0.9655479 |
| Sex | 0.0033263 | 0.0002099 | 0.7355648 | 0.6201332 | 0.7856016 | 0.0029238 | 0.2393334 | 0.8858669 | 0.8315003 | 0.9480492 |
| Time | 0.7787762 | 0.6387887 | 0.7979451 | 0.985093 | 0.9891684 | 0.3927782 | 0.7962797 | 0.9249937 | 0.8682986 | 0.7851587 |
| Treatment x Sex | 0.0231753 | 0.0038285 | 0.7735549 | 0.7062672 | 0.8562966 | 0.0221698 | 0.3806776 | 0.9503899 | 0.8718976 | 0.9615912 |
| Treatment x Time | 0.9523562 | 0.3188409 | 0.005725 | 0.1310247 | 0.0007997 | 0.1785108 | 0.0207779 | 0.0003592 | 0.0013978 | 0.0009511 |
| Sex x Time | 0.0284921 | 0.0046222 | 0.7514858 | 0.7203167 | 0.7388765 | 0.0243486 | 0.3572669 | 0.828601 | 0.7991901 | 0.844763 |
| Treatment x Sex x Time | 0.0219819 | 0.0291174 | 0.4149199 | 0.2902698 | 0.5106371 | 0.0402861 | 0.9054835 | 0.0246207 | 0.2952842 | 0.3765894 |
|  |  |  |  |  |  |  |  |  |  |  |
| **Fig. 7** | **Corticosterone** | **Serotonin** |  |  |  |  |  |  |  |  |
| # of samples/group | 4-5 | 4-5 |  |  |  |  |  |  |  |  |
| Treatment | 0.4821925 | 0.2998519 |  |  |  |  |  |  |  |  |
| Sex | 0.7377408 | 0.6768469 |  |  |  |  |  |  |  |  |
| Time | 0.000001 | 0.340173 |  |  |  |  |  |  |  |  |
| Treatment x Sex | 0.8552723 | 0.9155727 |  |  |  |  |  |  |  |  |
| Treatment x Time | 0.0701431 | 0.6909109 |  |  |  |  |  |  |  |  |
| Sex x Time | 0.0133689 | 0.8447312 |  |  |  |  |  |  |  |  |
| Treatment x Sex x Time | 0.4369431 | 0.7806204 |  |  |  |  |  |  |  |  |

**Table E2. Mouse antibodies used for immunophenotyping by flow cytometry.**

| **Antibodies Used** | **Cat No.** | **Clone** | **Source** |
| --- | --- | --- | --- |
| Alexa Fluor 488 anti-mouse CD24 antibody | 101816 | M1/69 | BioLegend |
| L/D510 |  | - |  |
| Brilliant Violet 605 anti-mouse CD45 antibody | 103155 | 30-F11 | BioLegend |
| Brilliant Violet 650 anti-mouse I-A/I-E antibody | 107641 | M5/114.15.2 | BioLegend |
| Brilliant Violet 785 anti-mouse CD11c antibody | 117336 | N418 | BioLegend |
| APC anti-mouse CD64 (FcγRI) antibody | 139306 | X54-5/7.1 | BioLegend |
| Alexa Fluor 700 anti-mouse Ly-6G antibody | 127622 | 1A8 | BioLegend |
| APC/Cyanine7 anti-mouse/human CD11b antibody | 101226 | M1/70 | BioLegend |
| PE anti-mouse CD193 (CCR3) antibody | 144506 | J073E5 | BioLegend |
| PR/Cyanine7 anti-mouse CD3ε antibody | 100320 | 145-2C11 | BioLegend |
| PE-CF594 rat anti-mouse Siglec-F | 562757 | E50-2440 | BD Biosciences |

**Table E3. Mouse gene-specific qRT-PCR primers used in this study.**

| **Gene symbol** | **Forward Primer Sequence (5’-3’)** | **Reverse Primer Sequence (5’-3’)** |
| --- | --- | --- |
| *Ccl2* | 5’-TTAAAAACCTGGATCGGAACCAA-3’ | 5’-GCATTAGCTTCAGATTTACGGGT-3’ |
| *Ccl8* | 5’-CTGGGCCAGATAAGGCTCC-3’ | 5’-CATGGGGCACTGGATATTGTT-3’ |
| *Muc5ac* | 5’-CCATGCAGAGTCCTCAGAACAA-3’ | 5’-TTACTGGAAAGGCCCAAGCA-3’ |
| *Rn18s* | 5’-GTAACCCGTTGAACCCCATT-3’ | 5’-CCATCCAATCGGTAGTAGCG-3’ |
