# Supplementary material for: Acute HDM exposure shows time-of-day and sex-based differences in the severity of lung inflammation and circadian clock disruption
Source: J Allergy Clin Immunol Glob. 2023 Jul 24;2(4):100155. doi: 10.1016/j.jacig.2023.100155 (PMC10509939; doi:10.1016/j.jacig.2023.100155)
Supplement: Supplementary Figures E1-E12 [file mmc3.docx]

**Supplemental Figures**


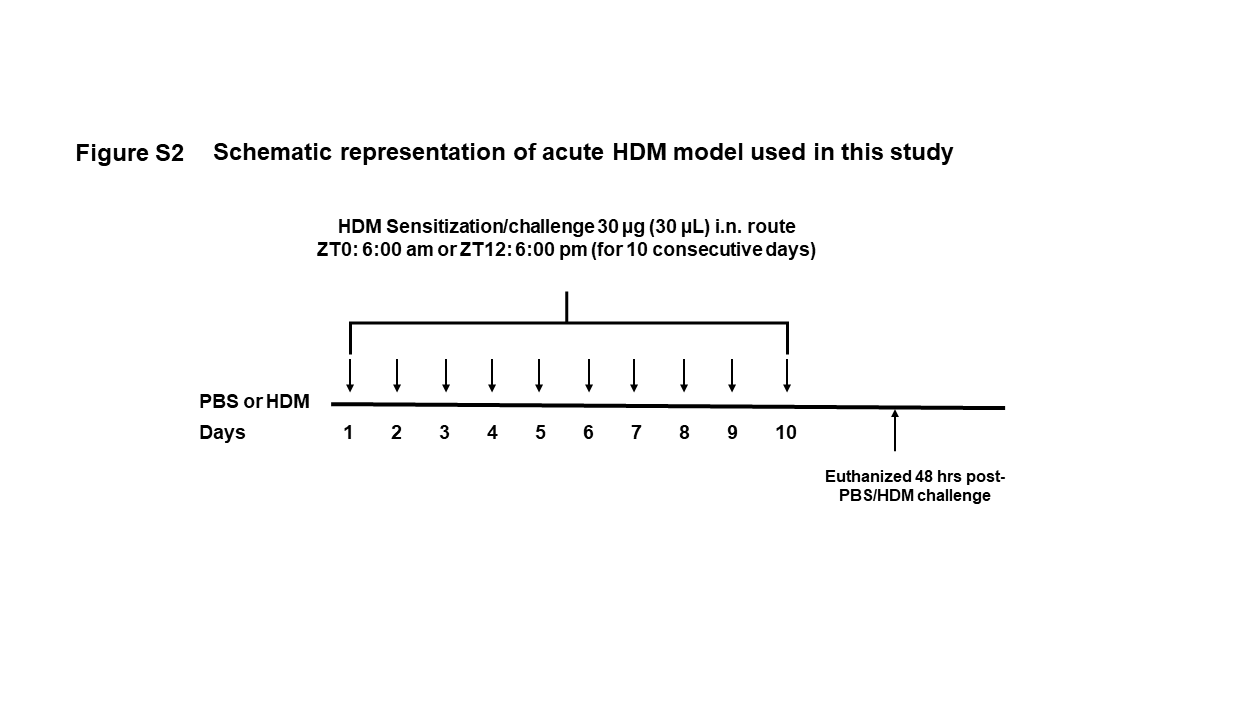


**Fig. E1. Schematic representation of acute HDM model used in this study.** C57BL/6J (2-3 months old, female, and male) mice were sensitized/challenged via the intranasal (i.n.) route (30 µg HDM in 30 µL) or sterile 1X PBS (30 µL) control for 10 days under mild anesthesia using 5% isoflurane. Serum, lung tissues, and BAL fluid were collected 48 hours post-last exposure for biochemical and molecular analysis.


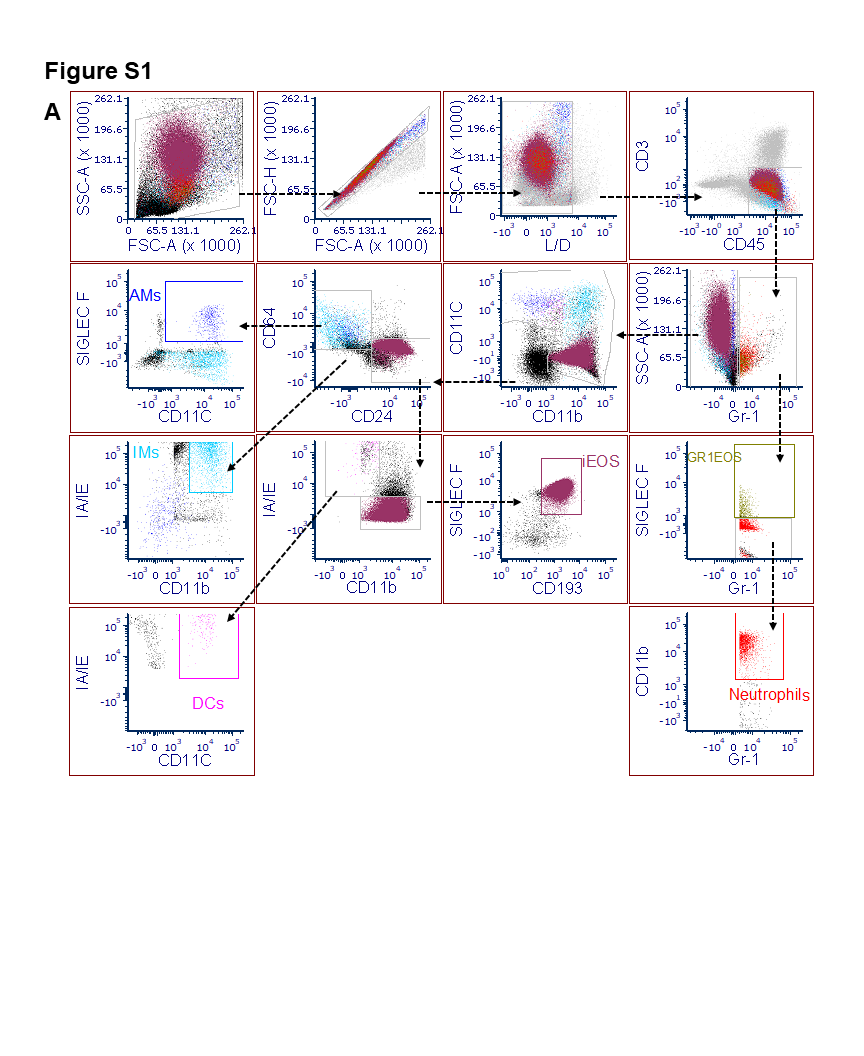

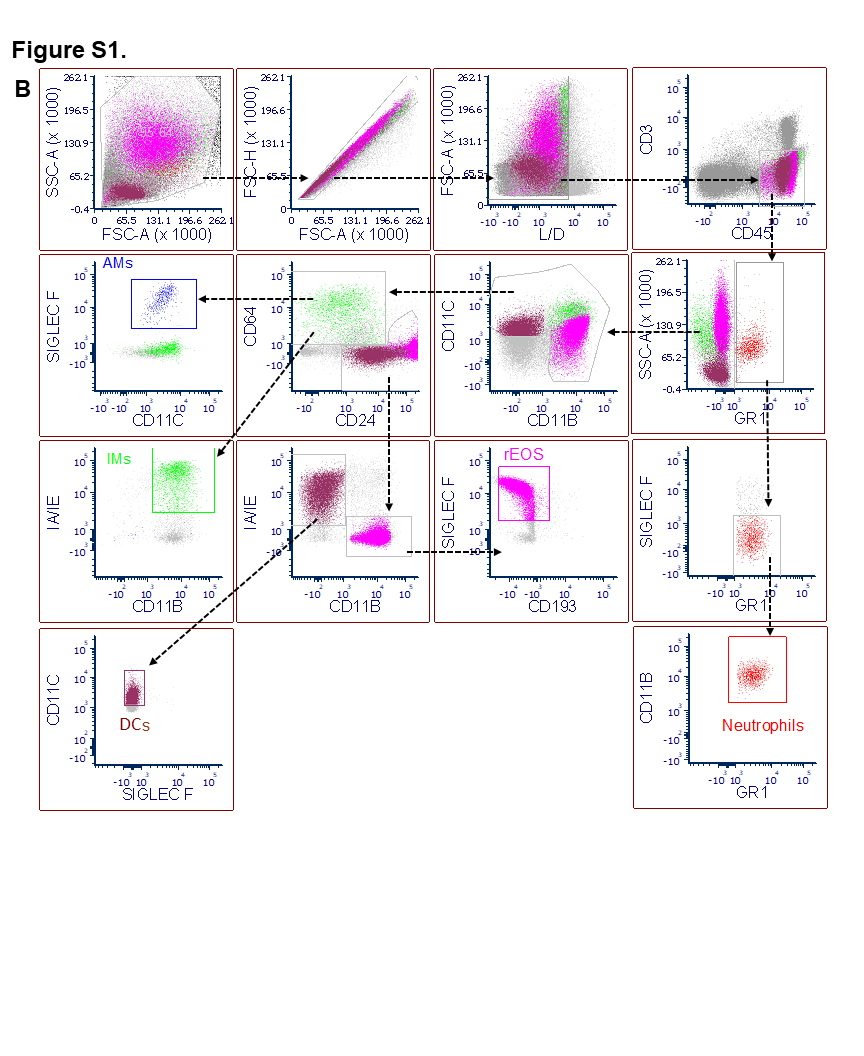


**Fig E2. Representative dot plots of a multicolor flow cytometry panel were used to identify myeloid cell subsets in the mouse.** (**A**) Bronchoalveolar lavage fluid and (**B**) single-cell suspension from enzymatically digested lung tissues (left lobe) of acute (10 d) PBS and HDM exposed mice at ZT0 and ZT12 were analyzed by flow cytometry. A custom design validated multicolor (11-color) antibody panel was used to identify myeloid cell subsets such as mononuclear phagocytic cells (macrophages, dendritic cells) and granulocytes (neutrophils and eosinophil subtypes) in this study.


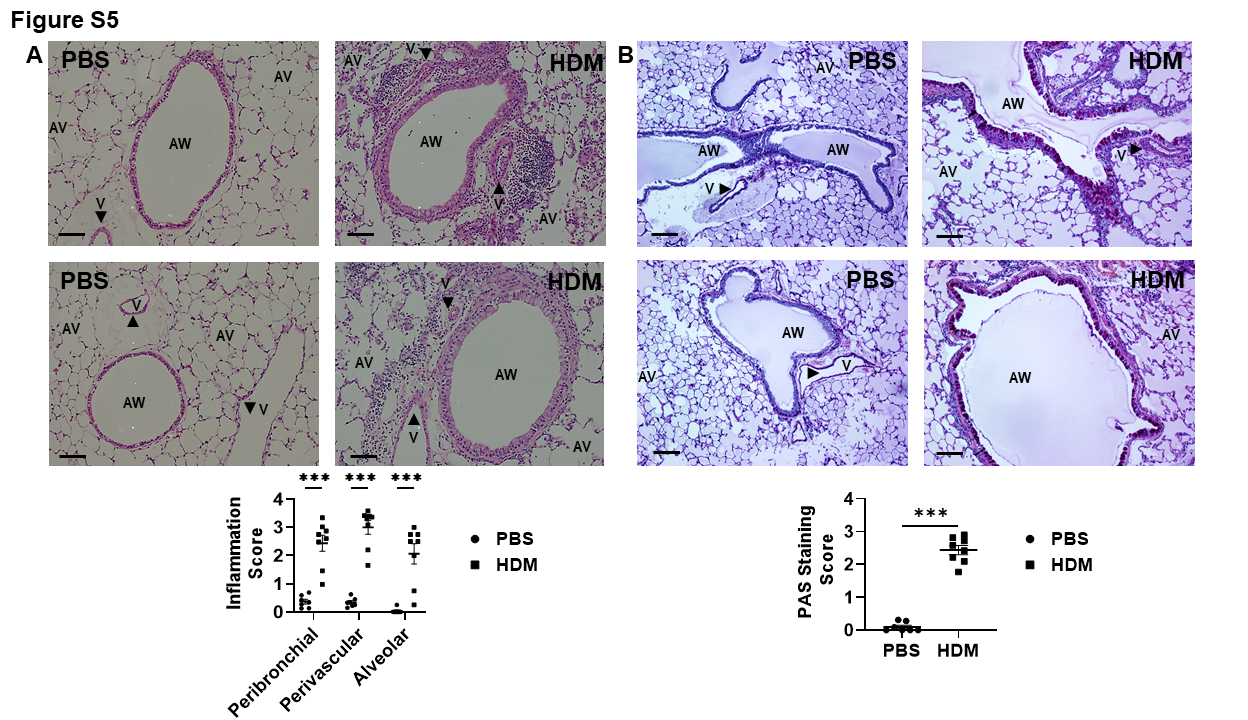


**Fig E3. Histological evaluation of acute HDM exposure showing increased lung inflammation and mucus production in mice.** Representative (**A**) Hematoxylin and Eosin (H&E) and (**B**) Periodic Acid Schiff’s-stained lung sections showing difference in the degree of lung inflammation (peribronchial [airway], perivascular and alveolar regions) and mucus production from acute 10d PBS and HDM exposed mice (combined females and males) are provided. Graph shows the average lung inflammation scores and PAS staining scores using the scoring criteria described in the materials and methods section in a blinded manner. Scale bar (100 µm). Data are shown as mean ± SEM (n=7-8/group; combined data from 3-4 females and males/group). ****P* < 0.001, compared to their respective control (PBS).


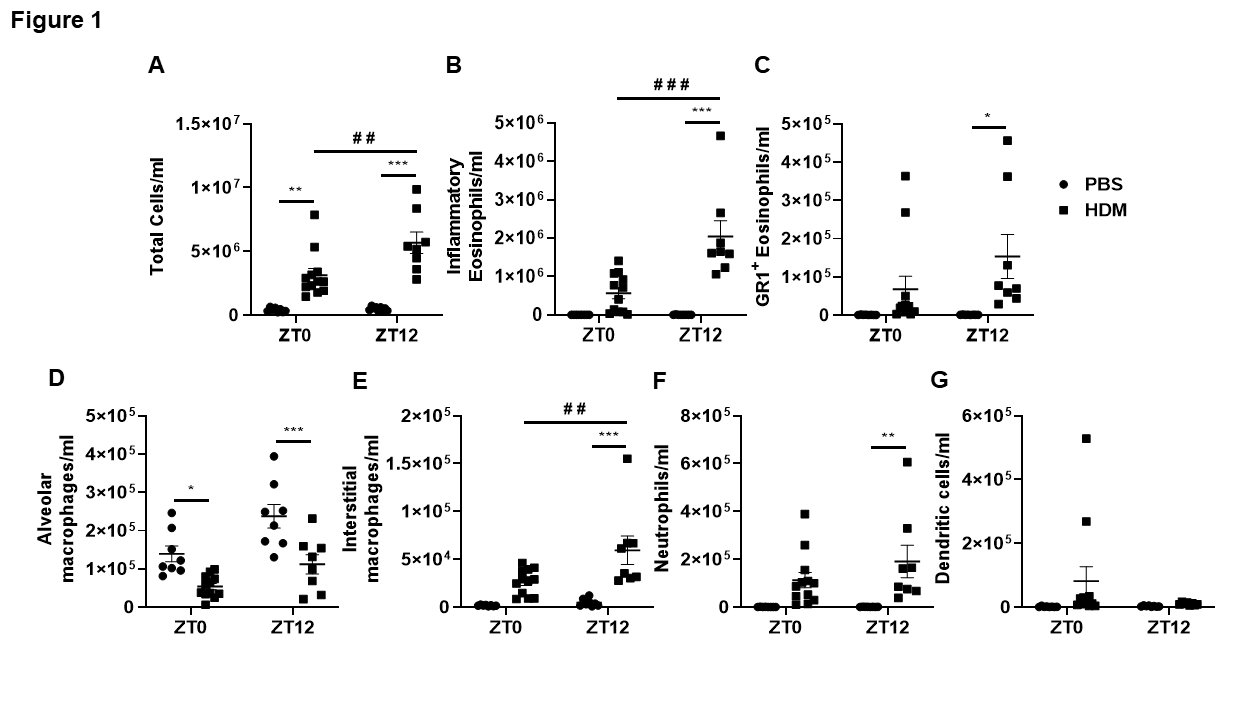


**Fig E4.** Myeloid cells in BAL fluid show time-of-day response to acute HDM exposure analyzed by flow cytometry. (**A**)Total cell counts in BAL fluid were determined using Countess II FL automated cell counter using Trypan blue staining. Myeloid cell types: (**B**) inflammatory Eosinophils (iEOS), (**C**) GR1^+^ Eosinophils (GR1^+^ EOS), (**D**) Alveolar Macrophages (AMs), (**E**) Interstitial Macrophages (IMs), (**F**) Neutrophils, and (**G**) Dendritic cells (DCs) from BAL fluid of acute (10 d) PBS and HDM exposed mice at ZT0 and ZT12 were analyzed by flow cytometry. Data are shown as mean ± SEM (n=8-12/group [combined female + male]). **P* < 0.05, ***P* < 0.01, ****P* < 0.001, compared to respective control (PBS) at ZT0 or ZT12; ^# #^*P* < 0.01, ^# # #^*P* < 0.001, compared to HDM at ZT0 vs. ZT12.


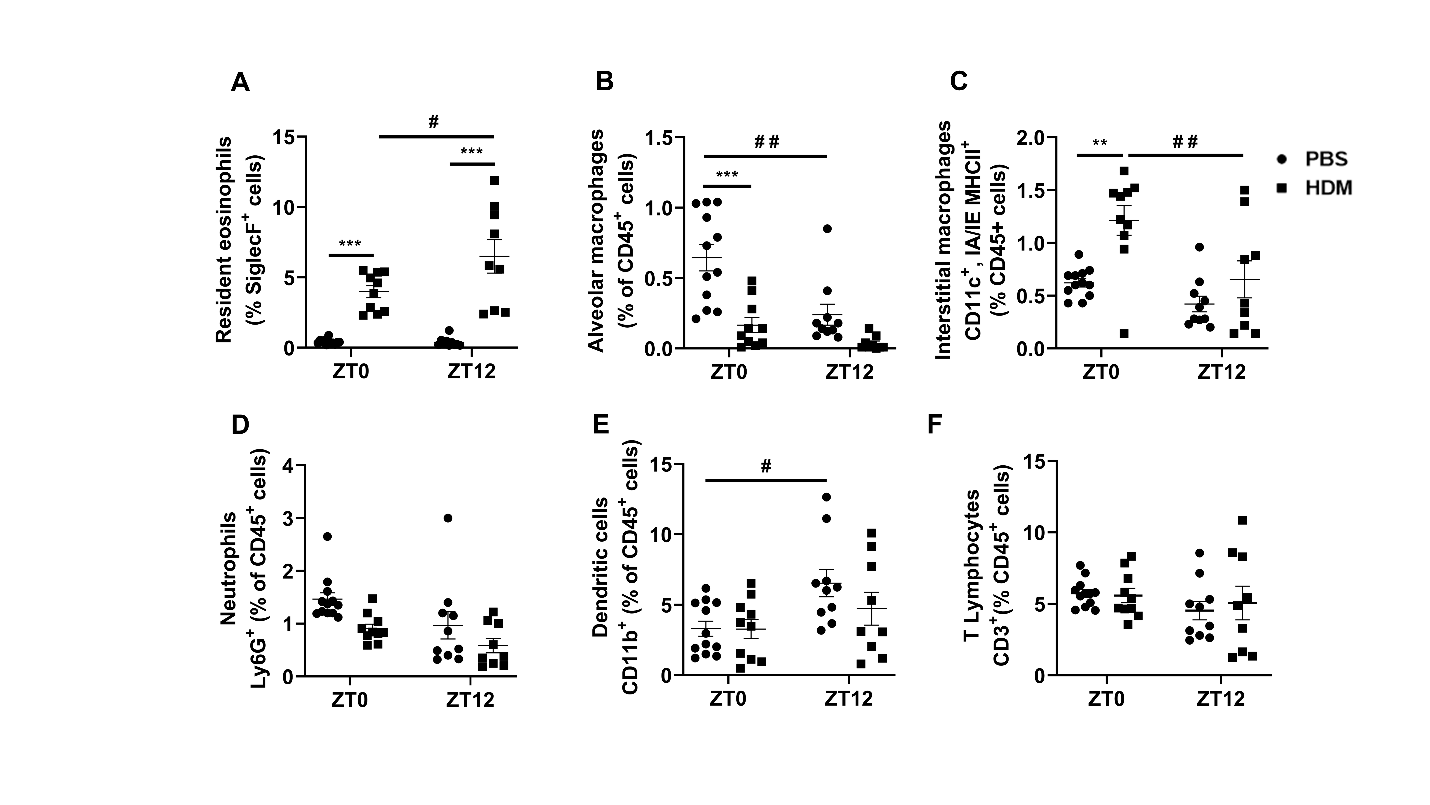


**Fig E5.** Myeloid cell infiltration in lung tissues shows a time-of-day response to acute HDM exposure analyzed by flow cytometry. Myeloid cell types: (**A**) Resident Eosinophils (rEOS), (**B**) Alveolar Macrophages (AMs), (**C**) Interstitial Macrophages (IMs), (**D**) Neutrophils, (**E**) Dendritic cells (DCs) and (F) T lymphocytes from lung tissue of acute (10 d) PBS and HDM exposed mice at ZT0 and ZT12 were analyzed by flow cytometry. Data are shown as mean ± SEM (n=9-12/group [combined female + male]). ***P* < 0.01, ****P* < 0.001, compared to respective control (PBS) at ZT0 or ZT12; ^#^ *P* < 0.05, ^# #^ *P* < 0.01, compared to HDM at ZT0 vs. ZT12.


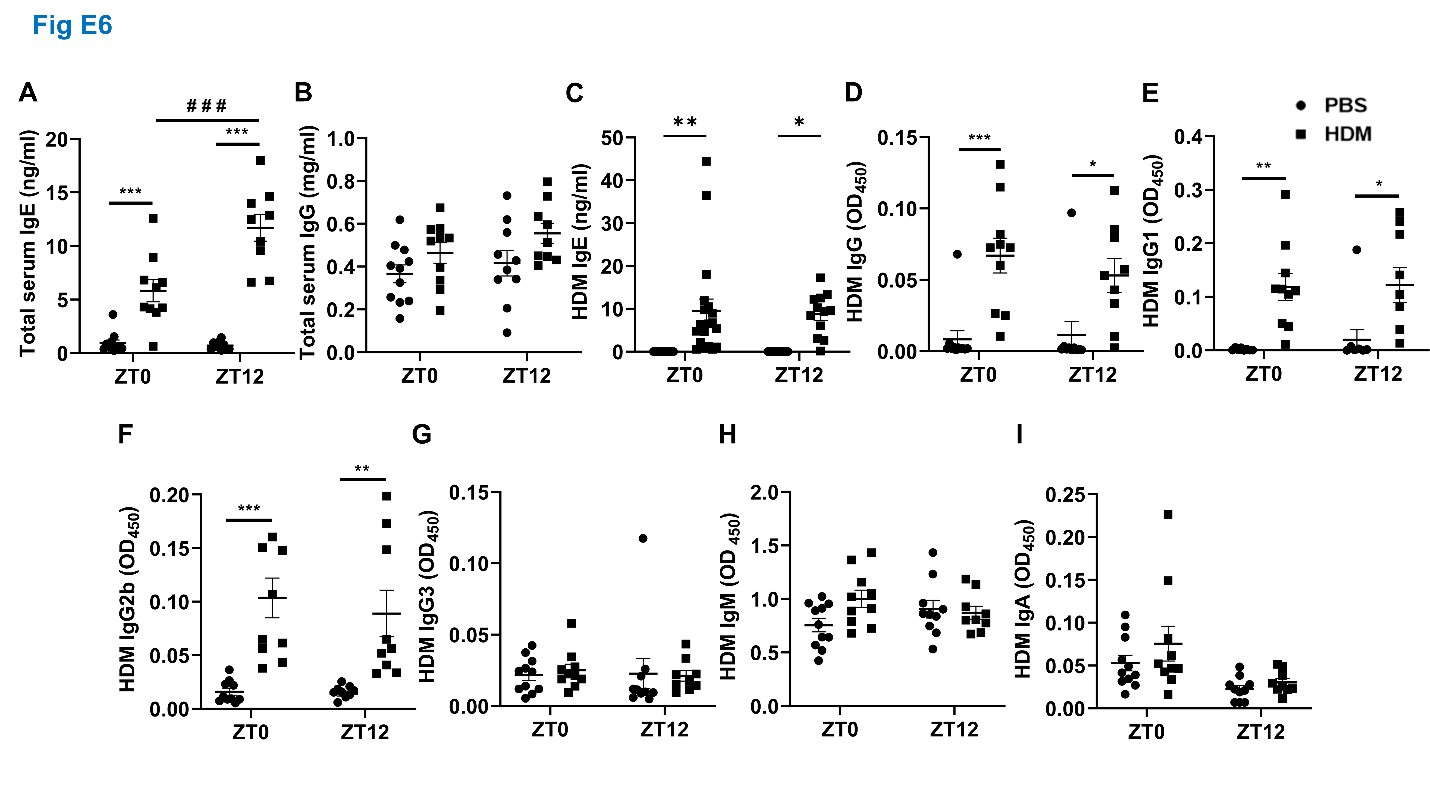
**Fig E6.** Serum total and HDM-specific immunoglobulin responses in acute HDM exposure. (**A**) Total IgE, (**B**) Total IgG, (**C**) HDM-specific IgE, (**D**) HDM-specific IgG, (**E**) HDM-specific IgG1, (**F**) HDM-specific IgG2b, (**G**) HDM-specific IgG3, (**H**) HDM-specific IgA and (**I**) HDM-specific IgM in the serum of acute (10 d) PBS and HDM exposed mice at ZT0 and ZT12 were determined by ELISA. Data were expressed as absorbance at 450 nm or ng/ml or mg/ml. Data are shown as mean ± SEM (n=9-11/group [combined female + male]). **P* < 0.05, ***P* < 0.01, ****P* < 0.001, compared to respective control (PBS) at ZT0 or ZT12; ^# # #^ *P* < 0.001, compared to HDM at ZT0 vs. ZT12.


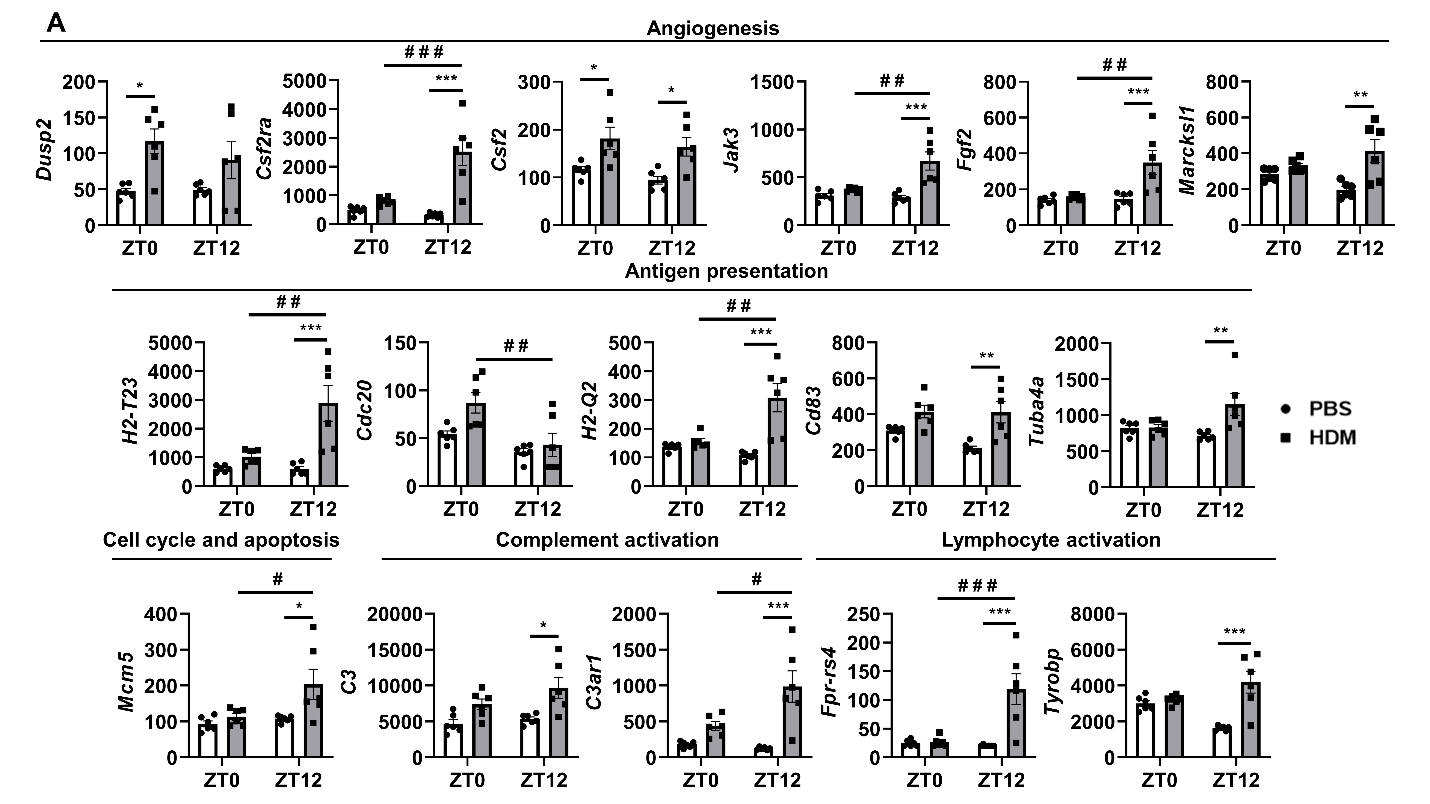


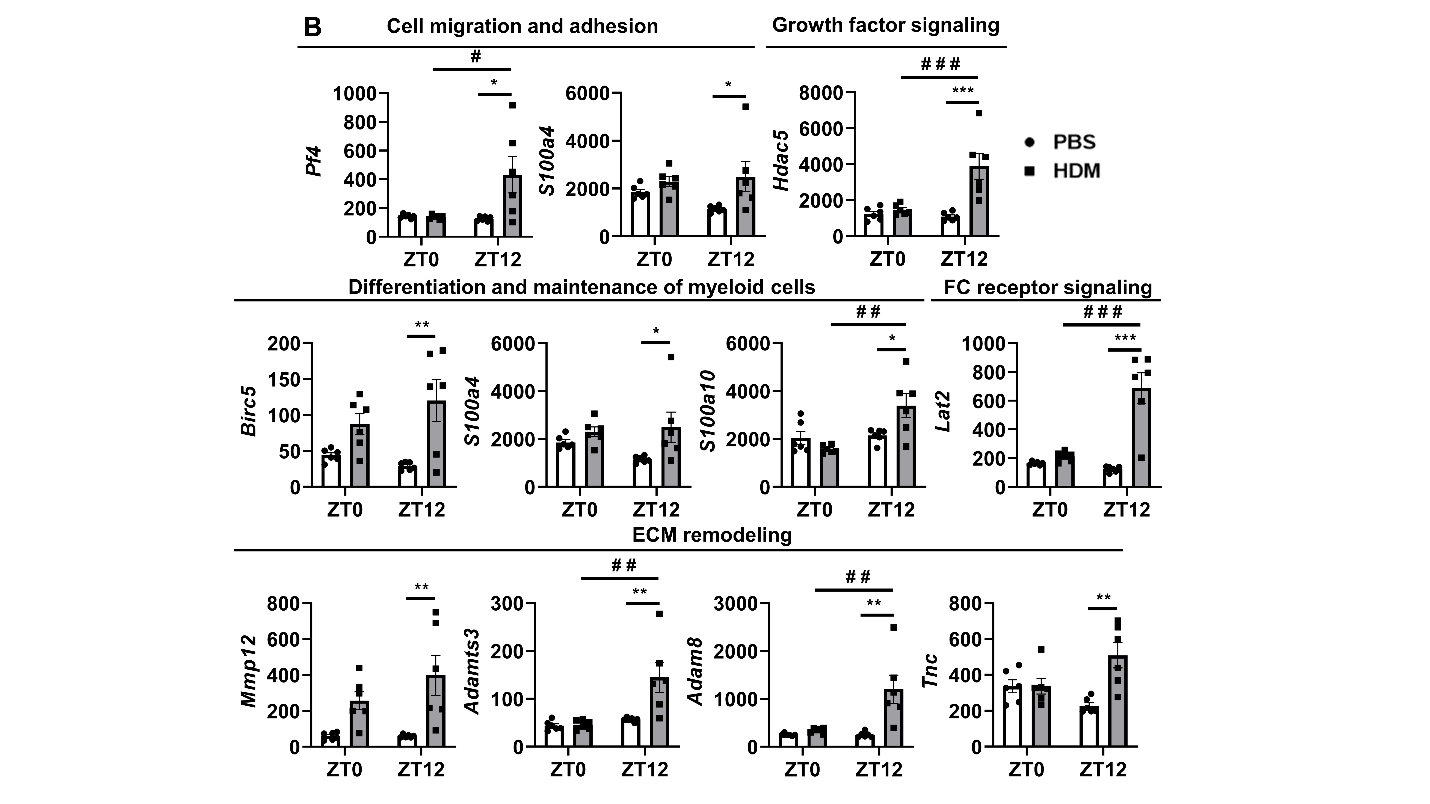


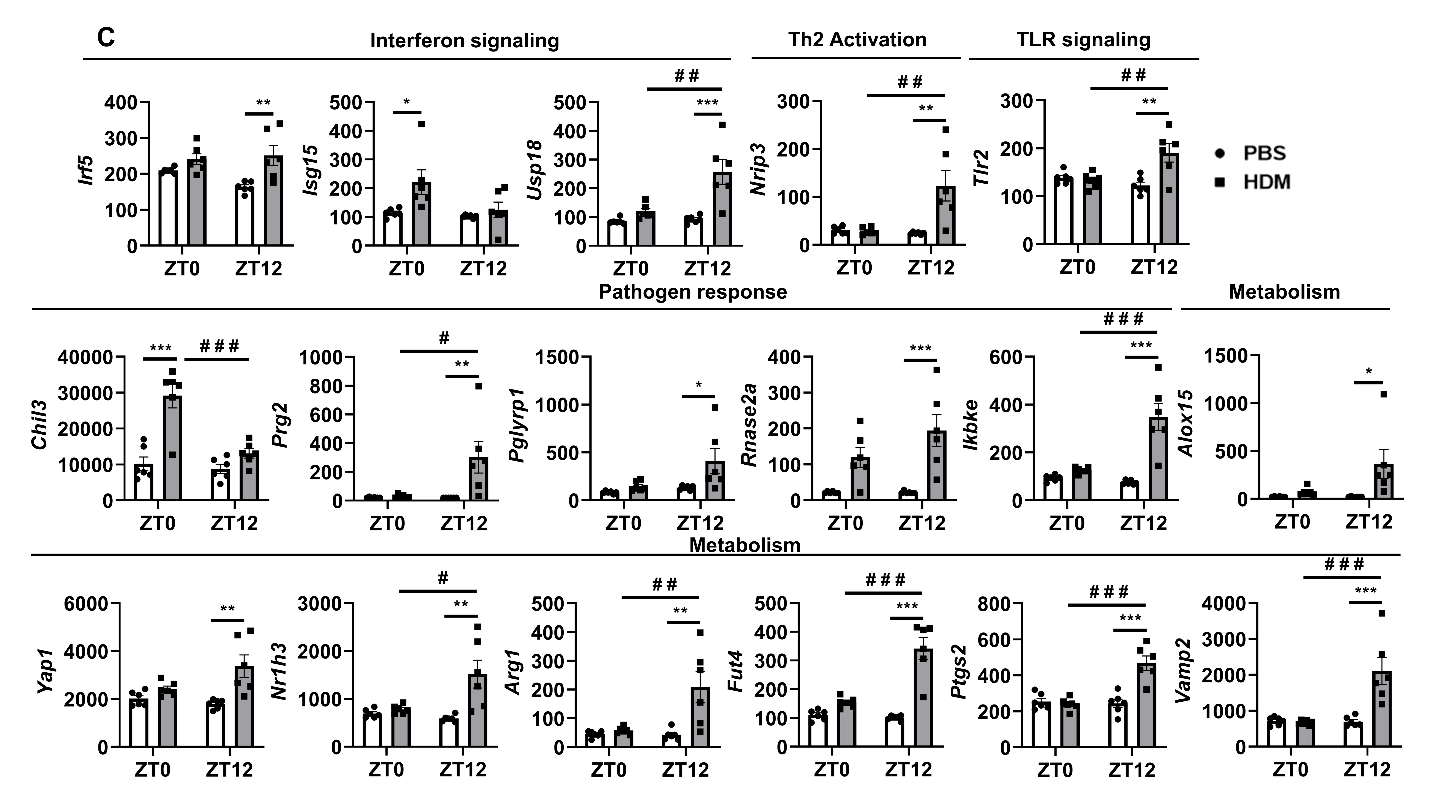


**Fig E7. Acute HDM exposure shows a time-of-day variation in the gene expression of key myeloid immunity target genes analyzed by NanoString**. Total RNA was isolated from the lungs of acute PBS and HDM-exposed mice at ZT0 and ZT12. We performed NanoString analysis using a mouse myeloid immunity panel in a nCounter SPRINT Profiler. Normalized RNA counts were analyzed using the nSolver analysis software (version 4.0). Genes that belong to the following annotations: (**A**) Angiogenesis, Antigen presentation, cell cycle and apoptosis, complement activation and lymphocyte activation, (**B**) Cell migration and adhesion, Chemokine signaling, Differentiation and maintenance of myeloid cells, FC receptor signaling, Growth factor signaling, ECM remodeling and T cell activation signaling, (**C**) Interferon signaling, Th2 activation, TLR signaling, Pathogen response, and Metabolism that showed significant upregulation in HDM exposed group compared to PBS control at ZT0 or ZT12 based on normalized counts data. White and grey bars in the graphs represent PBS and HDM groups respectively at ZT0 and ZT12. Data are shown as mean ± SEM (n=6 [3 females + 3 males]/group; **P* < 0.05, ***P* < 0.01, ****P* < 0.001, compared to respective control (PBS) at ZT0 or ZT12; ^#^*P* < 0.05, ^# #^*P* < 0.01, ^# # #^*P* < 0.01, compared to PBS or HDM at ZT0 vs. ZT12.


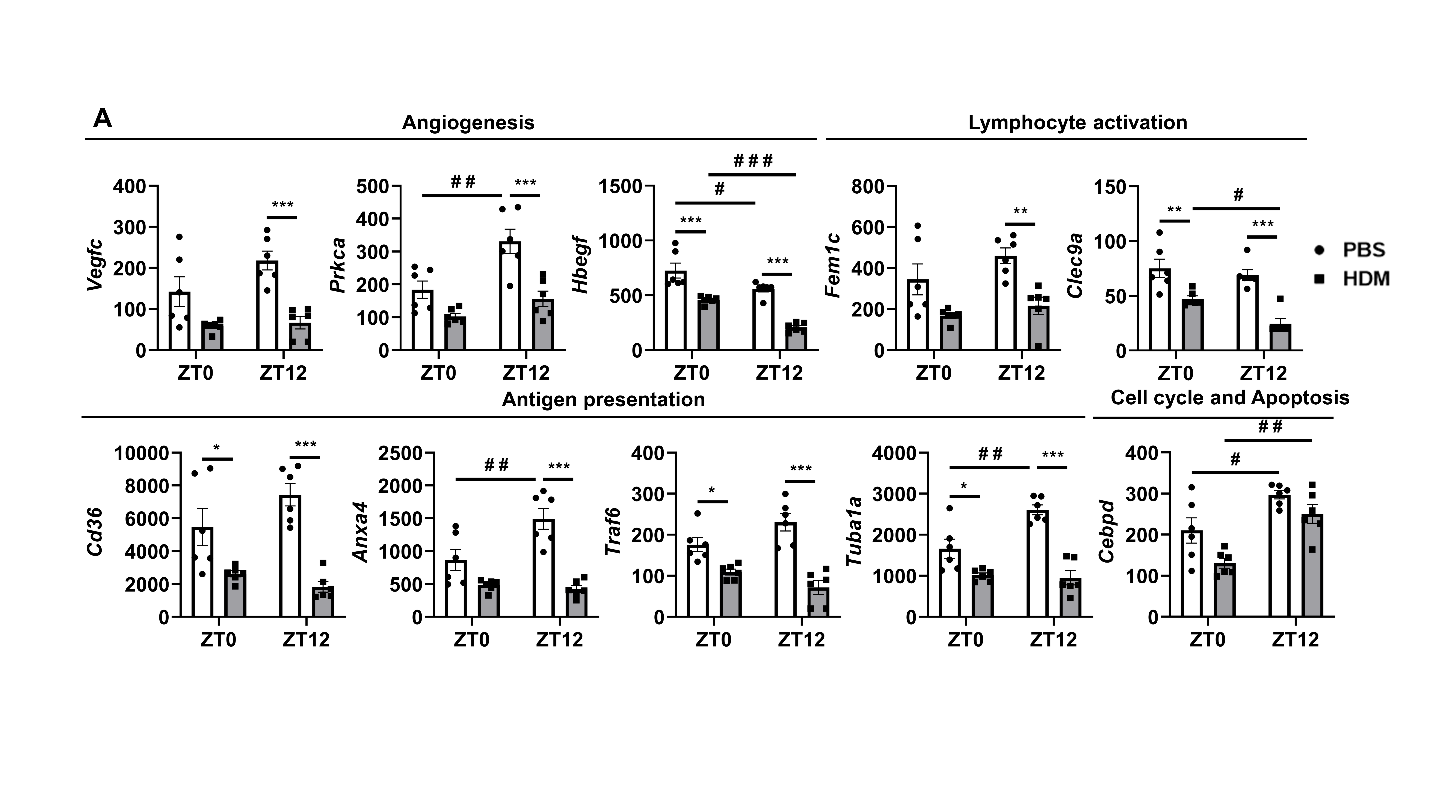


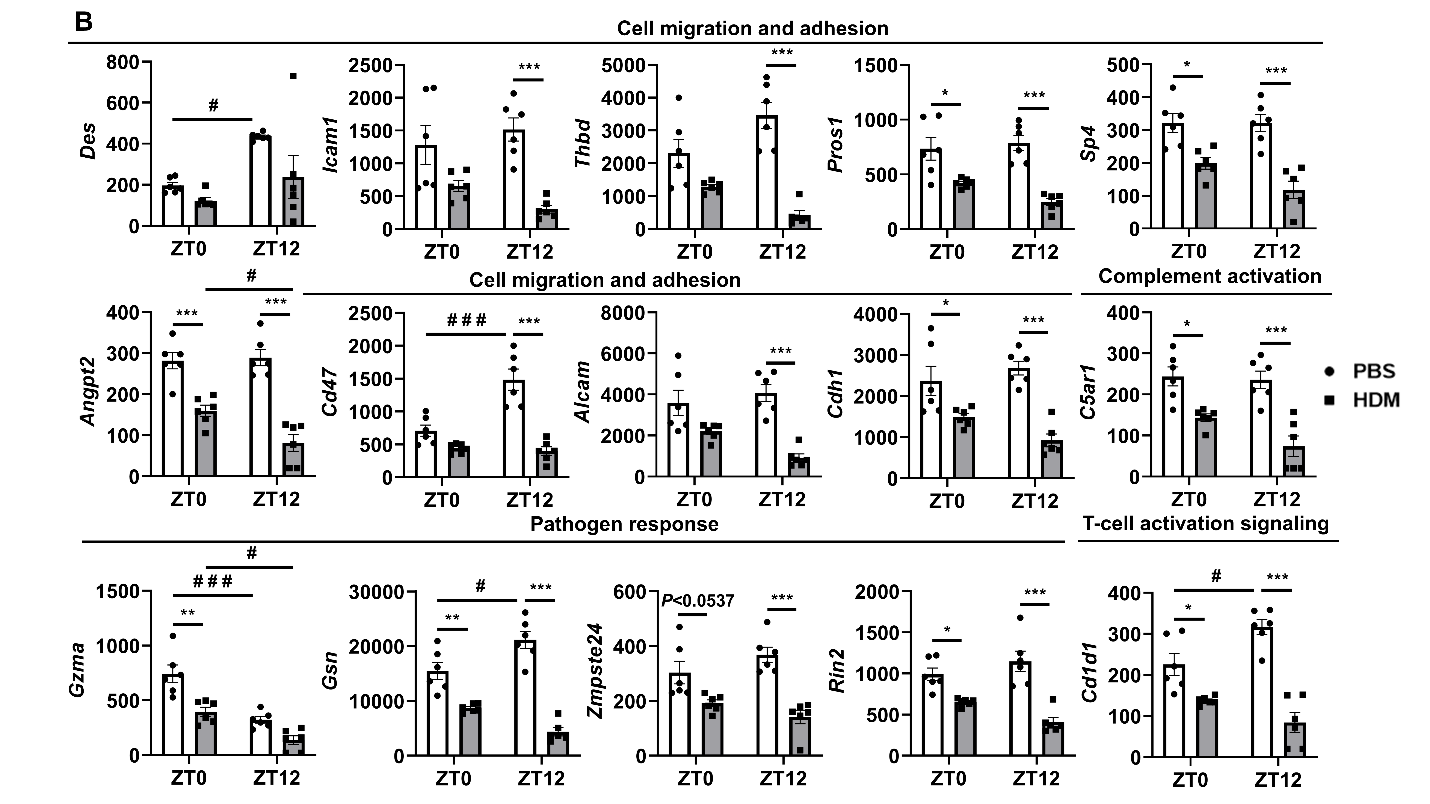


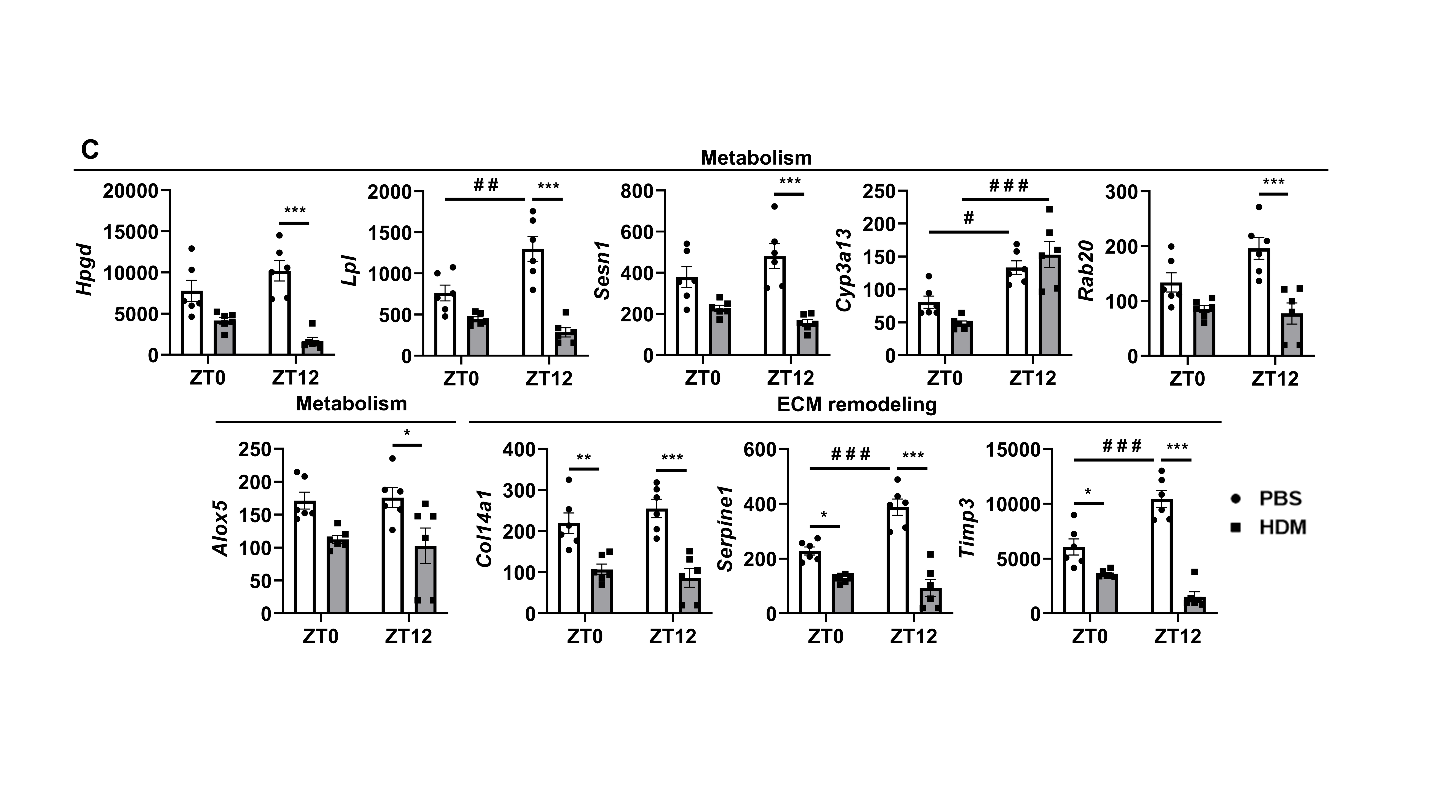


**Fig E8. Acute HDM exposure shows a time-of-day variation in the downregulation of key myeloid immunity target gene expression analyzed by NanoString**. Total RNA was isolated from the lungs of acute PBS and HDM-exposed mice at ZT0 and ZT12. We performed NanoString analysis using a mouse myeloid immunity panel in a nCounter SPRINT Profiler. Normalized RNA counts were analyzed using the nSolver analysis software (version 4.0). Genes that belong to the following annotations: (**A**) Angiogenesis, Lymphocyte activation, Antigen presentation, and cell cycle and apoptosis (**B**) Cell migration, and adhesion, Complement activation, Pathogen response, and T-cell activation signaling (**C**) Metabolism, and ECM remodeling that showed significant downregulation in HDM exposed group compared to PBS control at ZT0 or ZT12 based on normalized counts data. White and grey bars in the graphs represent PBS and HDM groups respectively at ZT0 and ZT12. Data are shown as mean ± SEM (n=6 [3 females + 3 males]/group; **P* < 0.05, ***P* < 0.01, ****P* < 0.001, compared to respective control (PBS) at ZT0 or ZT12; ^#^*P* < 0.05, ^# #^*P* < 0.01, ^# # #^*P* < 0.01, compared to PBS or HDM at ZT0 vs. ZT12.


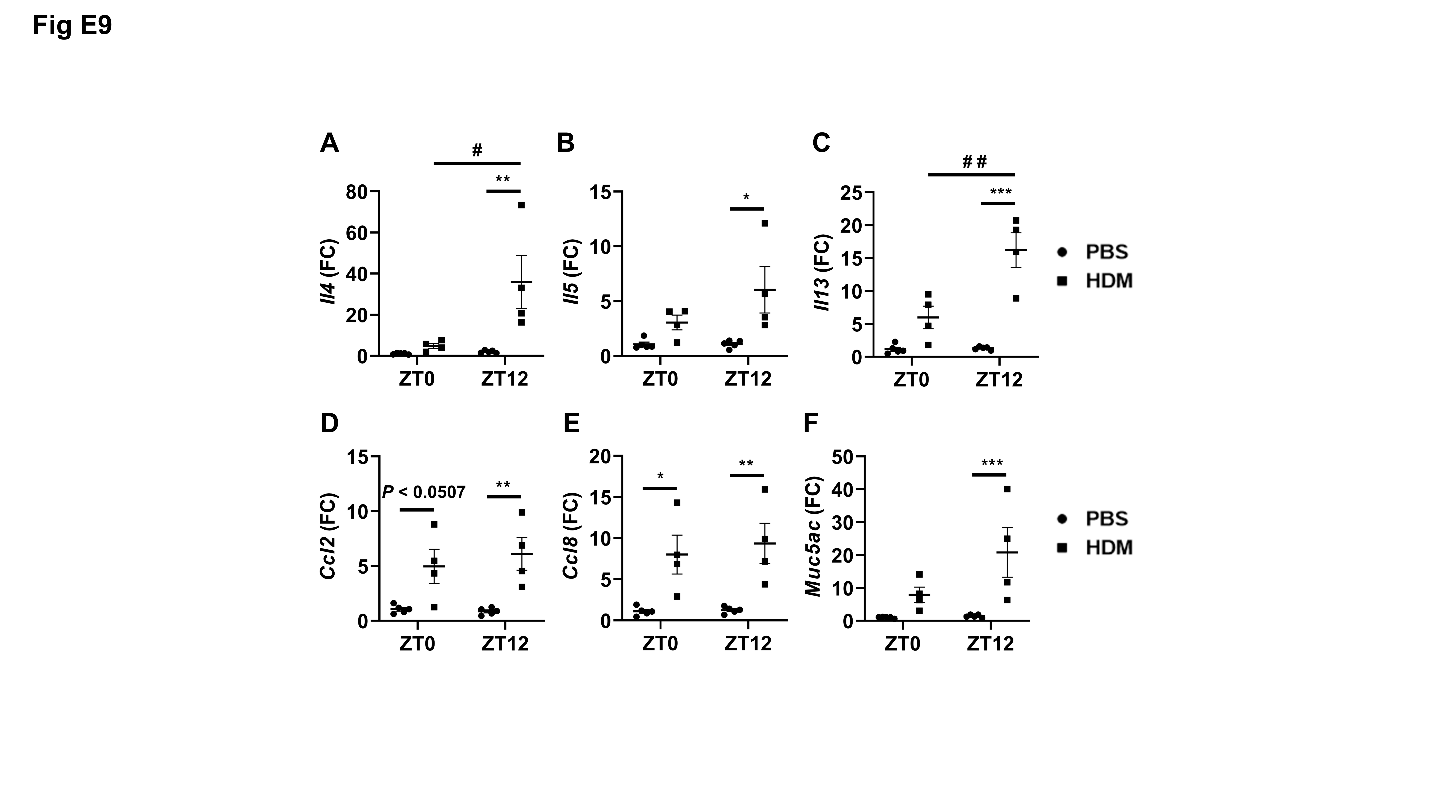


**Fig E9.** Acute HDM exposure affects the circadian gating of cytokines/chemokines and mucin genes and shows a time-of-day response in the lungs. Total RNA was isolated from the lungs of acute PBS and HDM-exposed mice at ZT0 and ZT12. (**A-C**) Gene expression of cytokines (interleukins: *il4, il5,* and *il13*), (**D-E**) chemokines (*ccl2*, and *ccl8*), and (**F**) mucin (*muc5ac*) genes were determined (relative to 18S rRNA) by qRT-PCR analysis and results are presented in relation to gene expression changes at ZT0 PBS exposed mice. Data are shown as mean ± SEM (n=4-5/group [females]; **P* < 0.05, ***P* < 0.01, ****P* < 0.001 compared to respective control (PBS) at ZT0 or ZT12; ^#^*P* < 0.05, ^# #^*P* < 0.01, compared to HDM at ZT0 vs. ZT12.


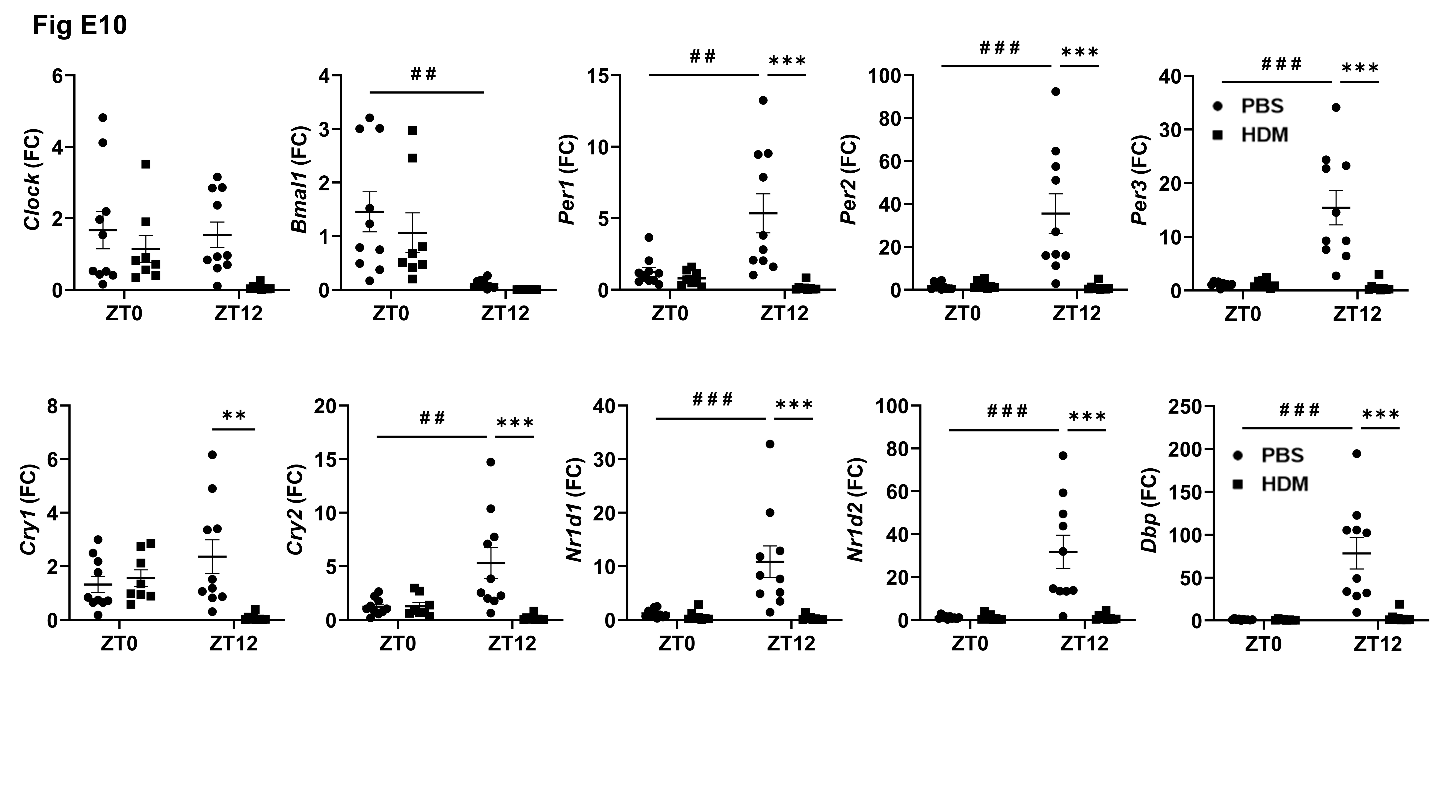


**Fig E10. Circadian clock-controlled genes show a time-of-day response following acute HDM exposure in the lung.** (**A-B**) C57BL/6J mice females and males were exposed to HDM or PBS at ZT0 and ZT 12 for 10 days and euthanized 48 hours after the last HDM exposure. Gene expression of core clock-controlled genes (*Clock, Bmal1, Per1, Per2, Per3, Cry1, Cry2, Nr1d1, Nr1d2*, and *Dbp*) was determined (relative to 18S rRNA) by qPCR analysis and the results are presented in relation to expression changes at ZT0 PBS exposed mice. Data are shown as mean ± SEM (n=4-5/group [females]; n=4-5/group [males] combined). ***P* < 0.01, ****P* < 0.001, compared to respective control (PBS) at ZT0 or ZT12; ^# #^*P* < 0.01, ^# # #^*P* < 0.001, compared to HDM or PBS at ZT0 vs. ZT12.


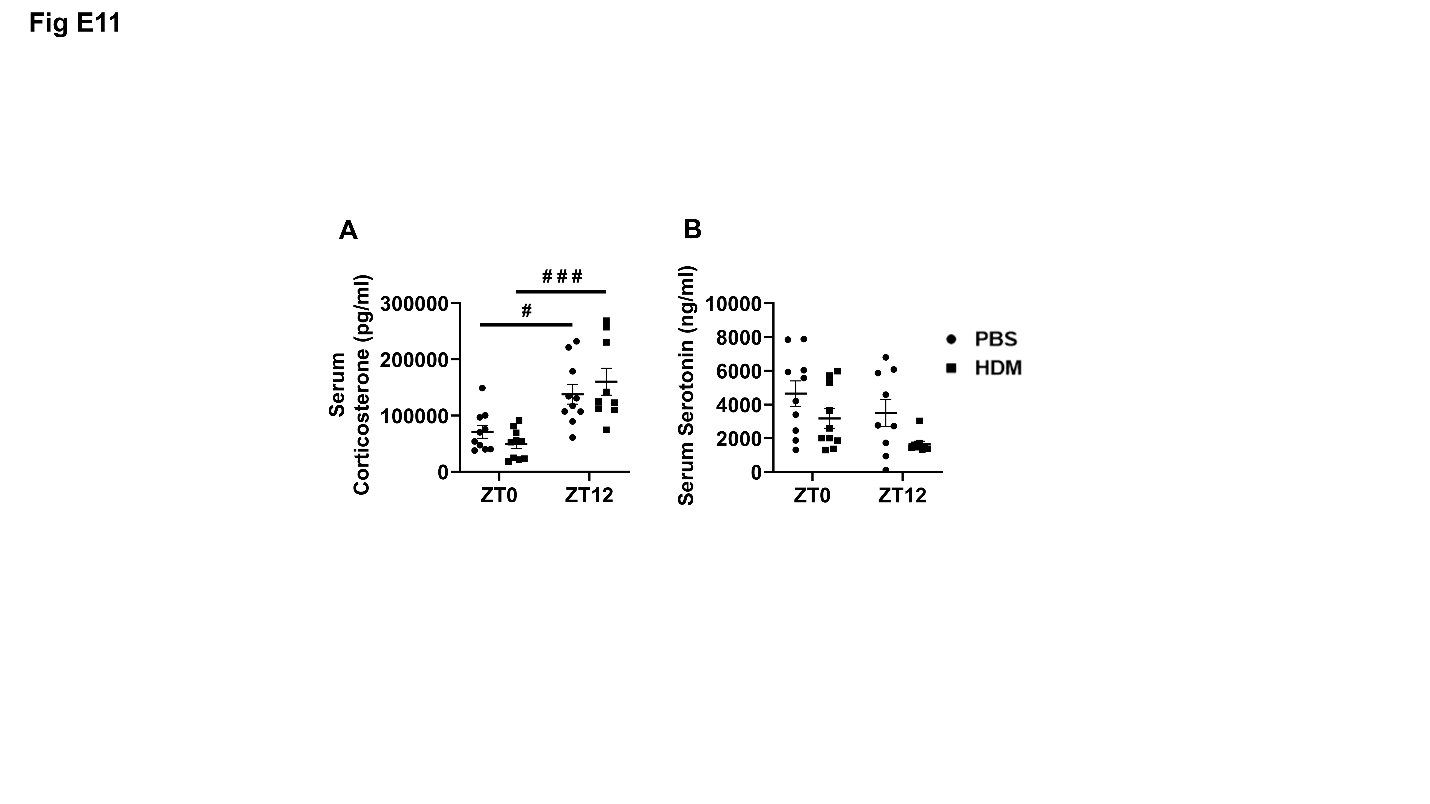


**Fig E11. Serum stress hormones corticosterone but not serotonin show a time-of-day difference in acute PBS and HDM-exposed mice.** Serum levels of (**A**) corticosterone and (**B**) serotonin were measured in acute (10 d) PBS and HDM-exposed females and males [combined] mice using commercially available competitive immunoassay. Data were expressed as pg/ml and ng/ml for corticosterone and serotonin, respectively. Data are shown as mean ± SEM (n=9-10/group [combined female + male]). ^#^ *P* < 0.05, ^# # #^ *P* < 0.001, compared to PBS or HDM at ZT0 vs. ZT12.


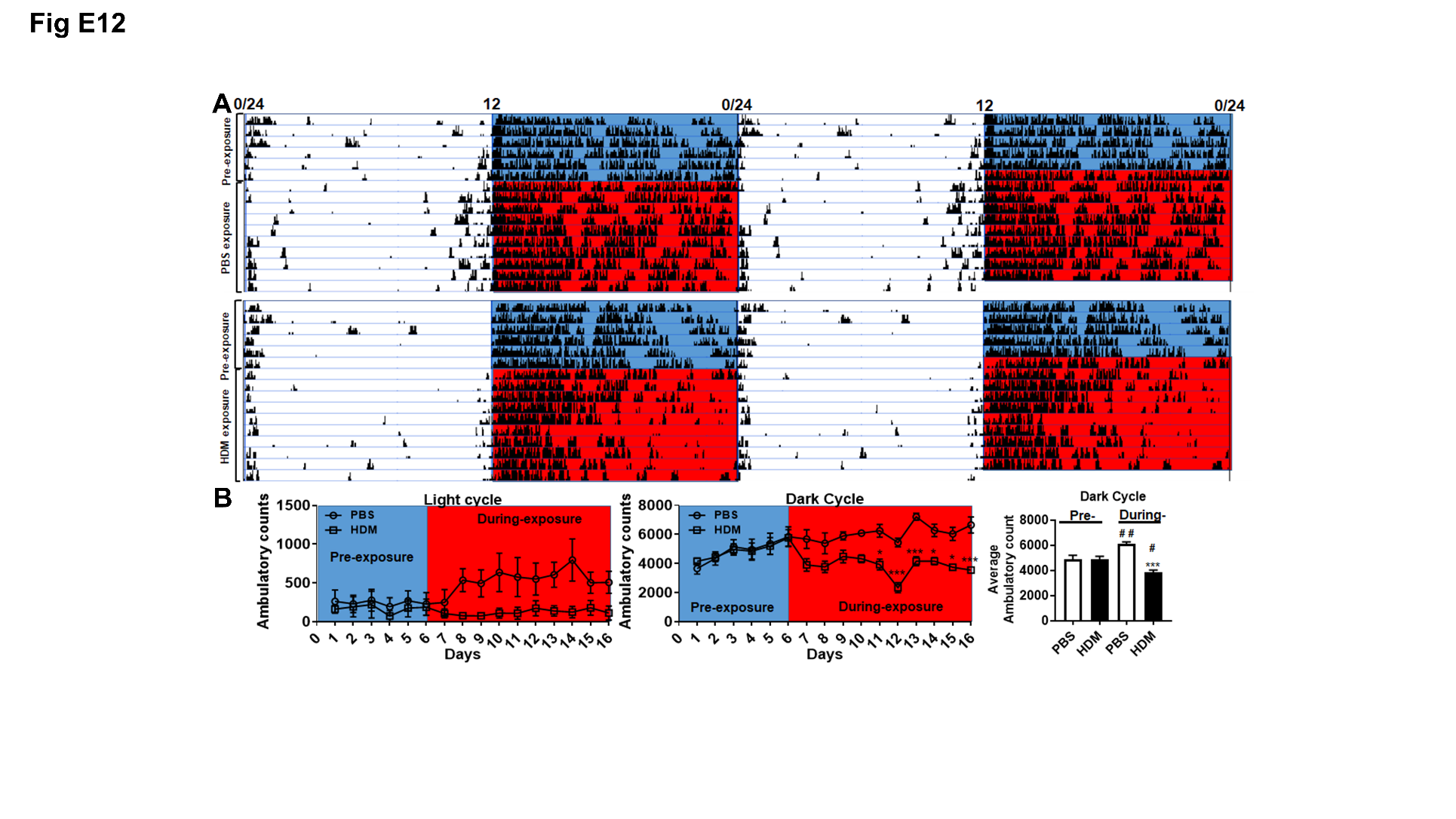


**Fig. E12. Effect of acute HDM exposure on circadian rhythms of wheel-running activity.** (**A**) Representative double-plotted actograms of mice exposed to acute 10 d PBS or HDM (30 μg) at ZT12. Mice were entrained to a 12:12 L:D cycle throughout this period. Blue shaded region indicates the dark phase 6 days before PBS or HDM challenge. Red-shaded regions indicate 10 days during acute PBS or HDM exposure. (**B**) Wheel running activity in L:D was plotted as ambulatory counts in Light and dark cycles across the 16 days period. Allergen challenge significantly reduced wheel running activity of mice during the dark cycle compared to PBS treated group. Data are mean ± SEM, n=3-4/group. * *P* < 0.05; *** *P* < 0.001 vs. PBS group; ^#^ *P* < 0.05; ^# #^ *P* < 0.01 pre-exposure vs during HDM exposure comparison.
